# The RNA Template Channel of the RNA-Dependent RNA Polymerase as a Target for Development of Antiviral Therapy of Multiple Genera within a Virus Family

**DOI:** 10.1371/journal.ppat.1004733

**Published:** 2015-03-23

**Authors:** Lonneke van der Linden, Laia Vives-Adrián, Barbara Selisko, Cristina Ferrer-Orta, Xinran Liu, Kjerstin Lanke, Rachel Ulferts, Armando M. De Palma, Federica Tanchis, Nesya Goris, David Lefebvre, Kris De Clercq, Pieter Leyssen, Céline Lacroix, Gerhard Pürstinger, Bruno Coutard, Bruno Canard, David D. Boehr, Jamie J. Arnold, Craig E. Cameron, Nuria Verdaguer, Johan Neyts, Frank J. M. van Kuppeveld

**Affiliations:** 1 Department of Medical Microbiology, Radboud Institute for Molecular Life Sciences, Radboud University Medical Centre, Nijmegen, The Netherlands; 2 Laboratory of Virology and Chemotherapy, Rega Institute for Medical Research, University of Leuven, Leuven, Belgium; 3 Institut de Biologia Molecular de Barcelona (CSIC), Parc Científic de Barcelona, Barcelona, Spain; 4 AFMB UMR 7257, Aix-Marseille Université & CNRS, Marseille, France; 5 Department of Chemistry, Pennsylvania State University, University Park, Pennsylvania, United States of America; 6 Virology Division, Department of Infectious Diseases and Immunology, Faculty of Veterinary Medicine, Utrecht University, Utrecht, The Netherlands; 7 Abteilung Pharmazeutische Chemie, Institut für Pharmazie, Universität Innsbruck, Innsbruck, Austria; 8 Okapi Sciences NV, Heverlee, Belgium; 9 Unit of Vesicular and Exotic Diseases, Virology Department, CODA-CERVA, Veterinary and Agrochemical Research Centre, Brussels, Belgium; 10 Department of Biochemistry and Molecular Biology, Pennsylvania State University, University Park, Pennsylvania, United States of America; Purdue University, UNITED STATES

## Abstract

The genus *Enterovirus* of the family *Picornaviridae* contains many important human pathogens (e.g., poliovirus, coxsackievirus, rhinovirus, and enterovirus 71) for which no antiviral drugs are available. The viral RNA-dependent RNA polymerase is an attractive target for antiviral therapy. Nucleoside-based inhibitors have broad-spectrum activity but often exhibit off-target effects. Most non-nucleoside inhibitors (NNIs) target surface cavities, which are structurally more flexible than the nucleotide-binding pocket, and hence have a more narrow spectrum of activity and are more prone to resistance development. Here, we report a novel NNI, GPC-N114 (2,2'-[(4-chloro-1,2-phenylene)bis(oxy)]bis(5-nitro-benzonitrile)) with broad-spectrum activity against enteroviruses and cardioviruses (another genus in the picornavirus family). Surprisingly, coxsackievirus B3 (CVB3) and poliovirus displayed a high genetic barrier to resistance against GPC-N114. By contrast, EMCV, a cardiovirus, rapidly acquired resistance due to mutations in 3D^pol^. *In vitro* polymerase activity assays showed that GPC-N114 i) inhibited the elongation activity of recombinant CVB3 and EMCV 3D^pol^, (ii) had reduced activity against EMCV 3D^pol^ with the resistance mutations, and (iii) was most efficient in inhibiting 3D^pol^ when added before the RNA template-primer duplex. Elucidation of a crystal structure of the inhibitor bound to CVB3 3D^pol^ confirmed the RNA-binding channel as the target for GPC-N114. Docking studies of the compound into the crystal structures of the compound-resistant EMCV 3D^pol^ mutants suggested that the resistant phenotype is due to subtle changes that interfere with the binding of GPC-N114 but not of the RNA template-primer. In conclusion, this study presents the first NNI that targets the RNA template channel of the picornavirus polymerase and identifies a new pocket that can be used for the design of broad-spectrum inhibitors. Moreover, this study provides important new insight into the plasticity of picornavirus polymerases at the template binding site.

## Author Summary

Virus replication relies on multiplication of viral genomes by viral polymerases. For enteroviruses, a large group of clinically important human pathogens for which no antiviral therapy is available, this function is performed by 3D^pol^, the RNA-dependent RNA polymerase. 3D^pol^ is therefore an attractive target for novel antiviral strategies. Most polymerase inhibitors identified today are nucleoside analogs, a class of compounds that exert broad-spectrum activity but often suffer from off-target effects. Non-nucleoside inhibitors on the other hand, in general have a more narrow spectrum of activity and are more prone to resistance development because in most cases they bind the surface of the enzyme which is less conserved and structurally more flexible. In this study, we present the identification of GPC-N114 as a non-nucleoside inhibitor of 3D^pol^ with broad-spectrum antiviral activity against both enteroviruses and cardioviruses, which also belong to the picornavirus family. Remarkably, it acts by targeting the RNA template-primer binding site in the core of 3D^pol^, making GPC-N114 the first anti-picornaviral compound with this mechanism of action. Thus, the characterization of GPC-N114 has led to the identification of a novel drug-binding pocket in 3D^pol^ that can serve as a starting point for antiviral drug design.

## Introduction

The family *Picornaviridae* contains 12 genera, and includes many human and animal pathogens (reviewed in [1]). Among these is the genus *Enterovirus* which contains four human enterovirus species (HEV-A, -B, -C, -D), three human rhinovirus species (HRV-A, -B, -C), simian enterovirus, bovine enterovirus, and porcine enterovirus. The HEV species include poliovirus (PV), coxsackievirus (CV), echovirus, and several numbered enteroviruses (EV). PV is the cause of poliomyelitis, which can lead to acute flaccid paralysis. Enterovirus 71, a major cause of hand-foot-and-mouth disease, is also frequently associated with flaccid paralysis and is a growing concern due to major epidemics in Southeast Asia. Coxsackieviruses are the main cause of viral meningitis, conjunctivitis, herpangina, myocarditis, and pancreatitis. HRV infections manifest themselves in most cases as the relatively mild common cold, but can cause serious exacerbations in patients with asthma or chronic obstructive pulmonary disease (COPD). Other well-known picornavirus genera are *Hepatovirus*, which contains hepatitis A virus, *Aphthovirus*, which contains foot-and-mouth disease virus (FMDV), and *Cardiovirus*, which includes encephalomyocarditis virus (EMCV), Theiler's murine encephalomyelitis virus, and the recently discovered Saffold virus (SAFV), which, unlike the other cardioviruses, is a human-tropic virus.

Currently, the toolbox to control picornavirus infections consists solely of vaccines against PV, hepatitis A virus, and FMDV. Prevention of diseases caused by the non-polio enteroviruses through vaccination seems unachievable given the great number of serotypes, with about 30 coxsackieviruses, 30 echoviruses, 50 numbered enteroviruses, and more than 150 rhinoviruses [2]. With this in mind, current efforts are aimed at developing antiviral compounds with broad-spectrum activity, targeting a wide range of viruses within a genus or ideally even multiple genera. No specific antiviral drugs have yet been clinically approved for the treatment of enteroviruses or any other picornavirus.

Picornaviruses are non-enveloped RNA viruses with a single-stranded 7–8 kb RNA genome of positive polarity. Upon entry, the viral RNA is translated into a polyprotein which is proteolytically processed by viral proteases to release the structural proteins (VP1-4) and the non-structural proteins (2A-2B-2C-3A-3B-3C-3D^pol^ and in some genera L) as well as some stable precursors. The genome is replicated via a negative-sense RNA intermediate by 3D^pol^, the viral RNA-dependent RNA polymerase (RdRP). For this, 3D^pol^ uses the viral protein 3B, in this context usually termed VPg (viral protein genome-linked), as a primer.

The structure of 3D^pol^ has been studied extensively in the past decades. Crystal structures have been reported of 3D^pol^ of the enteroviruses CVB3, PV, EV71, HRV1B, HRV14, and HRV16, and of the aphthovirus FMDV [3–10]. These enzymes adopt a classical right hand architecture, with fingers, palm and thumb subdomains providing the correct geometrical arrangement of substrate molecules and metal ions at the polymerase active site for catalysis [11]. In addition, in all picornavirus RdRPs, the conformation of the right hand is closed by the connection of fingers and thumb sub-domains through the N-terminal portion of the protein and several loops protruding from fingers, named the fingertips. This connection leads to the formation of a completely encircled active site and largely restricts the inter-domain mobility. Three well-defined channels have been identified in the RdRP structures that allow access to the active site of the incoming rNTPs and the RNA template, as well as the exit path of the newly synthesized dsRNA product [11]. Seven conserved structural elements, termed motifs A–G, have been defined. Motifs A–E, located in the palm subdomain, are involved in nucleotide and nucleic acid binding as well as catalysis. Motifs F and G, located in the fingers, play a critical role in the binding of nucleoside triphosphates and RNA templates [12].

Given its critical role in genome replication, 3D^pol^ is regarded as an attractive target for antiviral drugs. Polymerase inhibitors can be subdivided into two classes. Most inhibitors identified so far fall under the class of nucleoside/nucleotide analogs, which compete with endogenous nucleotides and may act as chain terminators and/or mutagens. One example in this class is ribavirin which induces lethal mutagenesis [13]. The second class of polymerase inhibitors are the non-nucleoside inhibitors (NNI) which can have a variety of mechanisms of action, e.g., stabilizing the enzyme such that necessary conformational changes cannot take place [14]. Frequently, these NNIs bind enzyme surfaces which are more variable between viruses and consequently NNIs are more likely to have a restricted spectrum of activity. Also, the barrier to resistance is lower due to the greater tolerance to amino acid substitutions. A few NNIs active against picornaviruses have been discovered, but for most of them, the mechanism of action remains poorly characterized [15–22]. In this study, we report a novel NNI, GPC-N114 that, unlike most NNIs, binds the core of 3D^pol^ and possesses broad-spectrum activity against enteroviruses and cardioviruses. GPC-N114 represents the first picornavirus 3D^pol^ inhibitor that targets the template-binding site and reveals a novel pocket in 3D^pol^ that is amenable for broad-spectrum inhibition.

## Results

### GPC-N114 is an inhibitor of enterovirus and cardiovirus replication

**Fig 1:**
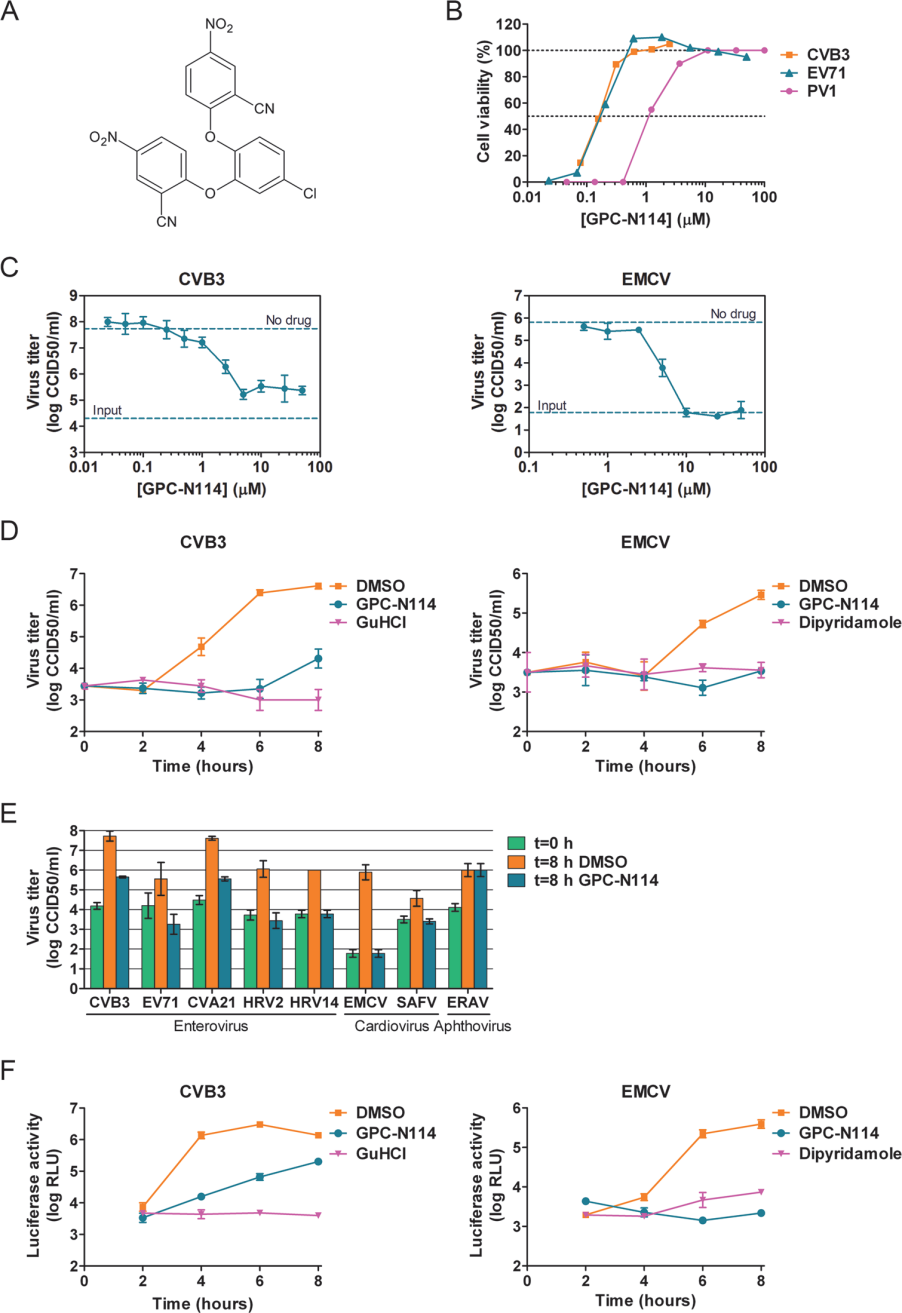
GPC-N114 inhibits picornavirus replication. (A) Structural formula of GPC-N114. (B) Representative dose-response curves of multicycle CPE-reduction assays for CVB3, PV1, and EV71. CPE was quantified by MTS assay at 3 d p.i. and is expressed as percentage of uninfected, untreated controls. (C, D) Antiviral activity of GPC-N114 against CVB3 and EMCV. BGM cells were infected with CVB3 (left panels) or EMCV (right panels) at an MOI of 0.1. Immediately after infection, GPC-N114 was added at the indicated concentrations (C) or at 10 μM (D). The enterovirus inhibitor guanidine hydrochloride (GuHCl) and the cardiovirus inhibitor dipyridamole were included as controls. Virus titers were determined by endpoint titration after 8 h (C) or at the indicated times p.i. (D). Experiments were performed in triplicate and mean values ± SD are depicted. (E) Antiviral activity of GPC-N114 against a range of picornaviruses. Cells were infected with the indicated viruses at an MOI of 0.5 after which 10 μM GPC-N114 was added. Virus titers were determined at 8 h p.i. Experiments were performed in triplicate and mean values ± SD are depicted. (F) GPC-N114 inhibits viral RNA replication. RNA of subgenomic replicons of CVB3 or EMCV was transfected into BGM cells. Subsequently, cells were treated either with 0.1% DMSO, 10 μM GPC-N114, 2 mM GuHCl, or 80 μM dipyridamole. Firefly luciferase levels were determined 2, 4, 6, and 8 h after RNA transfection to assess the level of replication and translation. Experiments were performed in triplicate and mean values ± SD are depicted.

We recently described a series of 5-nitro-2-phenoxybenzonitriles that inhibit *in vitro* enterovirus replication [23]. Further optimization of this class of molecules led to the identification of 2,2'-[(4-chloro-1,2-phenylene)bis(oxy)]bis(5-nitro-benzonitrile), hereafter referred to as GPC-N114 (**Fig. 1A**), with potent and selective *in vitro* antiviral activity against CVB3. This small molecule inhibits CVB3 replication in multicycle CPE-reduction antiviral assay with a 50% effective concentration (EC_50_) of 0.15 ± 0.02 μM (**Table 1**).

**Table 1:** Antiviral activity of GPC-N114.

Serotype	Species	Cell line	EC_50_ (μM)
Enterovirus			
EV71	HEV-A	RD	0.13 ± 0.05
CVA16	HEV-A	Hela H	0.21 ± 0.02
CVB3	HEV-B	Vero	0.15 ± 0.02
E11	HEV-B	BGM	0.93 ± 0.01
CVA21	HEV-C	Hela R19	0.94 ± 0.23
PV1	HEV-C	BGM	1.00 ± 0.60
PV2	HEV-C	BGM	0.88 ± 0.32
PV3	HEV-C	BGM	0.26 ± 0.01
EV68	HEV-D	Hela R19	1.44 ± 0.13
EV70	HEV-D	Hela R19	1.55 ± 0.28
HRV2	HRV-A	Hela R19	0.55 ± 0.03
HRV14	HRV-B	Hela R19	1.73 ± 0.25
Cardiovirus			
Mengovirus	EMCV	BGM	5.44 ± 0.49
Aphthovirus			
FMDV-O	FMDV	BHK-21	>25
FMDV-A	FMDV	BHK-21	>25
ERAV-1	ERAV	BGM	>100

Shown are mean values calculated from at least three experiments ± SD. The cytotoxicity values (CC_50_) were for Hela H cells 8.54 ± 0.42 μM, for Hela R19 cells 7.07 ± 0.38 μM, and for RD, BGM, and Vero cells >100 μM

To study its spectrum of activity, GPC-N114 was evaluated in similar multicycle antiviral assays with representatives of the different HEV and HRV species, as well as several other picornaviruses. All enteroviruses and rhinoviruses included in this study were found to be sensitive to the inhibitory effect of GPC-N114, with EC_50_ values ranging from 0.1 μM to 1.7 μM (**Fig. 1B and Table 1**). GPC-N114 also inhibited the replication of EMCV (strain mengovirus), a member of the genus *Cardiovirus* (EC_50_ = 5.4 ± 0.49 μM). By contrast, FMDV and equine rhinitis A virus (ERAV), two members of the genus *Aphthovirus*, proved insensitive to its inhibitory effect (tested with concentrations up to 25 μM and 100 μM, respectively).

To evaluate the antiviral effect of GPC-N114 in a single round of replication, CVB3 and EMCV, as representatives of the enterovirus and cardiovirus genus, were incubated in the presence of different concentrations of the compound and virus titers were determined at 8 h post infection (p.i.). Cell viability assays performed in parallel showed that GPC-N114 was not toxic at these concentrations (**S1A Fig.**). For CVB3, the maximal inhibitory effect was obtained with concentrations of 3 μM and higher, although replication was not fully abrogated (**Fig. 1C**). Replication of EMCV was fully inhibited at concentrations of 10 μM or higher (**Fig. 1C**). Therefore, a concentration of 10 μM was selected for all further cellular assays. Detailed analysis of the kinetics of CVB3 and EMCV replication at this concentration showed that GPC-N114 strongly delayed virus replication (**Fig. 1D and S1B Fig.**). Comparable results were observed for a panel of other enteroviruses (EV71, CVA21, HRV2, and HRV14) and cardioviruses (EMCV and SAFV) (**Fig. 1E and S1B Fig.**). Again, no antiviral activity was observed against the aphthovirus ERAV (**Fig. 1E**).

Together, these results demonstrate that GPC-N114 exerts broad-spectrum *in vitro* antiviral activity, inhibiting the replication of a representative panel of viruses belonging to the enterovirus and cardiovirus genera from the picornavirus family.

### GPC-N114 targets the RNA replication stage of the virus replication cycle

To identify the stage of the replication cycle that is targeted by GPC-N114, we used CVB3 and EMCV subgenomic replicons in which (a part of) the P1 coding region has been replaced with the firefly luciferase gene. Luciferase expression thus allows the quantification of translation and replication of the viral RNA. At 2 h post-transfection, a time point when no viral RNA replication has taken place yet [24], luciferase production from both replicons due to translation of incoming RNA was comparable in the presence and absence of GPC-N114 (**Fig. 1F**), suggesting that GPC-N114 does not affect translation. However, at later time points, luciferase levels were lower in GPC-N114-treated cells in comparison with mock-treated control cells for both the CVB3 and the EMCV replicon. In agreement with the single cycle assays, the EMCV replicon proved more sensitive to the inhibitory effect of GPC-N114 than the CVB3 replicon. These results indicate that GPC-N114 targets the viral RNA replication stage.

To determine whether the impaired replication in the presence of GPC-N114 is caused by a defect in the processing of the viral polyprotein, the viral proteins were visualized by metabolic labeling. CVB3-infected BGM cells were pulse-labeled with [^35^S]Met in the presence or absence of the compound between 5.5 and 6 h p.i., a time when translation of cellular, capped mRNAs is shut off by the virus [25]. The labeled proteins were analyzed by SDS-PAGE and autoradiography. The quantity and pattern of the viral proteins was similar for treated and untreated samples (**S2 Fig.**), demonstrating that GPC-N114 does not affect synthesis or processing of the viral polyprotein.

### EMCV variants resistant to GPC-N114 carry mutations in 3D^pol^

**Fig 2:**
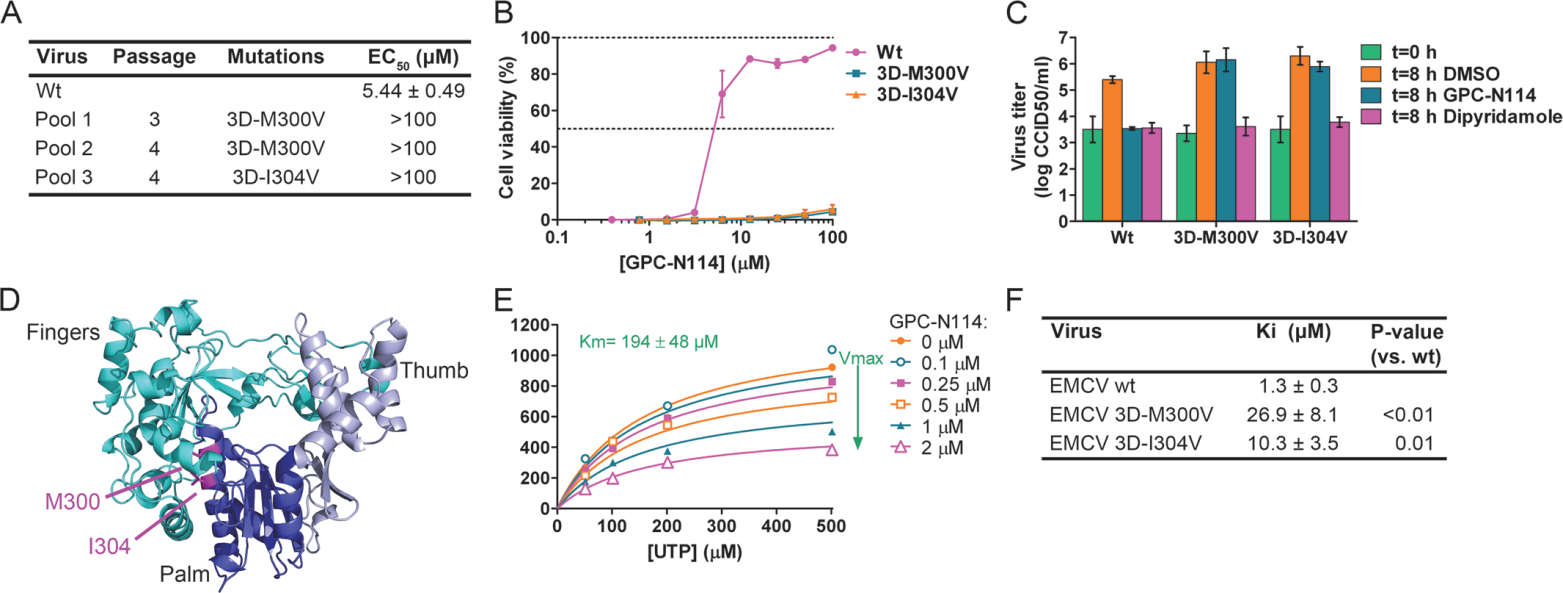
Mutations in EMCV 3D^pol^ confer resistance to GPC-N114. (A) Location of the mutations in GPC-N114-resistant EMCV pools and the EC_50_ of recombinant viruses as determined in a multicycle CPE-reduction assay. (B,C) Recombinant EMCV 3D^pol^ mutants are resistant to GPC-N114, as shown by multicycle CPE reduction assay (B) or single cycle growth curves (C). For technical details, see legend to Fig. 1. (D) Ribbon diagram of the three-dimensional structure of EMCV 3D^pol^ shown in the conventional orientation, as if looking into a right hand. The two residues mutated in GPC-N114-resistant EMCV (M300 and I304) are indicated in magenta. (E) GPC-N114 inhibits EMCV 3D^pol^ activity, but not by competing with UTP. Polymerase elongation activity in the presence of a range of concentrations of GPC-N114 was determined by measuring incorporation of [^3^H]UTP using poly(rA)/dT15 as template-primer. Michaelis-Menten plots of the initial velocity of EMCV 3D^pol^ elongation activity determined at different concentrations of UTP and GPC-N114 are depicted. The curves shown are based on a non-competitive mode of inhibition. (F) Inhibition constants of GPC-N114 on EMCV 3D^pol^ wt and mutants M300V and I304V. Inhibition constants (Ki) were calculated from at least three experiments performed as in (E) and values shown are mean ± SD. Statistical analysis was performed with unpaired Student’s *t*-test.

By serial passaging of virus in the presence of increasing concentrations of GPC-N114, we aimed to select for GPC-N114-resistant virus. For this, we used a protocol that previously allowed us to select virus variants resistant against a variety of compounds on different cell lines [26–28]. However, despite 32 passages on Vero cells and several attempts, no compound-resistant CVB3 isolates were obtained, indicating a high resistance barrier for this virus to GPC-N114. Also no resistant viruses were isolated on BGM cells. The possibility that the inability to obtain resistant CVB3 was due to the incomplete inhibition of replication by GPC-N114 (as indicated by single-cycle assays, though CPE was completely inhibited in multicycle assays), resulting in a too low selection pressure, seems unlikely because we also failed to isolate GPC-N114-resistant variants of PV1 (of which replication was completely blocked by GPC-N114 in a single-cycle assay, **S3 Fig.**) and E9. In contrast, three independent virus pools of EMCV showed phenotypic resistance after as few as three or four serial passages in the presence of suboptimal concentrations of the compound. Viral RNA was isolated from these virus pools and the regions coding for the non-structural proteins were sequenced. Interestingly, the three virus pools each contained a single missense mutation compared to wild-type (wt) virus. These mutations were located either at nucleotide 7115 (A7115G) or 7127 (A7127G) resulting in amino acid changes M300V and I304V in the viral RdRP, 3D^pol^, respectively (**Fig. 2A**).

To verify the causal relationship between these mutations and the resistance phenotype, the mutations were introduced into the EMCV cDNA clone pM16.1 and mutant viruses were generated. In the multicycle CPE-reduction assay, the EC_50_ dramatically increased from 5.44 ± 0.49 μM for EMCV wt to >100 μM for EMCV 3D-M300V and 3D-I304V, indicating that the mutants were resistant to the compound (**Fig. 2A and B**). Accordingly, while the production of infectious virus particles by wt virus was strongly inhibited in a single cycle assay, virus titers of the mutant viruses after 8 h were unaffected by the presence of 10 μM GPC-N114 (**Fig. 2C**). As expected, the mutations did not confer resistance to the cardiovirus inhibitor dipyridamole. These results demonstrate that the mutations in 3D^pol^ were indeed responsible for the resistance phenotype of the isolated virus pools.

The X-ray structure of EMCV 3D^pol^ has been recently reported [29]. It shows that the amino acids M300 and I304 are both located in structural motif B both on the same face of helix α10 (which is part of the palm subdomain of the polymerase), close to the active site (**Fig. 2D**). Both residues are buried in the core of the protein and their side chains are involved in hydrophobic interactions that stabilize the local structure.

Having identified GPC-N114 resistance mutations in EMCV 3D^pol^, we set out to test whether the corresponding mutations in CVB3 (i.e., 3D-I296V and 3D-M300V) would yield GPC-N114-resistant variants. Mutations were introduced in a CVB3 cDNA by site-directed mutagenesis and viruses were obtained by transfection of run-off transcripts in cells. The mutant viruses displayed similar increases in virus titer after 8 hours as CVB3 wt in the absence of compound, but did not show a compound-resistant phenotype (**S4A Fig.**). Also mutation S299T in CVB3 3D^pol^, which was previously shown to provide resistance against the 3D^pol^ inhibitor amiloride [19,22], failed to confer resistance to GPC-N114 (**S4B Fig.**).

### GPC-N114 inhibits *in vitro* polymerase activity

The location of the EMCV resistance mutations suggested that GPC-N114 targets the activity of 3D^pol^. To verify this, biochemical assays were performed using purified EMCV 3D^pol^ in the presence or absence of GPC-N114. Briefly, elongation of a dT15 primer on a poly(rA) template by recombinant enzyme was determined by measurement of the rate of incorporation of [^3^H]UTP. Elongation activity of EMCV 3D^pol^ on the poly(rA) template was inhibited by GPC-N114 in a dose-dependent manner (**Fig. 2E**), which is in line with the resistance to the compound mapping to 3D^pol^ in EMCV. Using the same template-primer and metal ion (i.e., Mg^2+^), GPC-N114 did not affect the elongation activity of the polymerase domain of the dengue virus (DENV) NS5 RdRP (**S5 Fig.**, IC_50_ > 100 μM), demonstrating that the inhibition of EMCV 3D^pol^ activity was not due to an unspecific interference with these components of the assay.

An inhibition constant (Ki) of 1.3 μM was calculated from the EMCV 3D^pol^ elongation activity measured at multiple UTP and inhibitor concentrations (**Fig. 2F**). **Fig. 2E** shows representative Michaelis-Menten plots of the results acquired with EMCV 3D^pol^. The data were fitted by non-linear regression assuming a competitive, noncompetitive, uncompetitive or mixed mode of inhibition. The model of noncompetitive inhibition provided the best fit, with the maximum velocity (Vmax) of the reaction declining with increasing concentrations of the compound. This indicates a noncompetitive mode of inhibition with respect to UTP (and presumably NTPs in general). Markedly higher inhibition constants were observed for the two resistant mutants (M300V, 26.9 μM; I304V, 10.3 μM) as compared to that for the wt enzyme (1.3 μM) (**Fig. 2F**). Thus, GPC-N114 inhibited EMCV 3D^pol^ elongation activity, which could be counteracted by mutations M300V and I304V.

### GPC-N114 binds 3D^pol^ at the site of the template acceptor nucleotide

**Fig 3:**
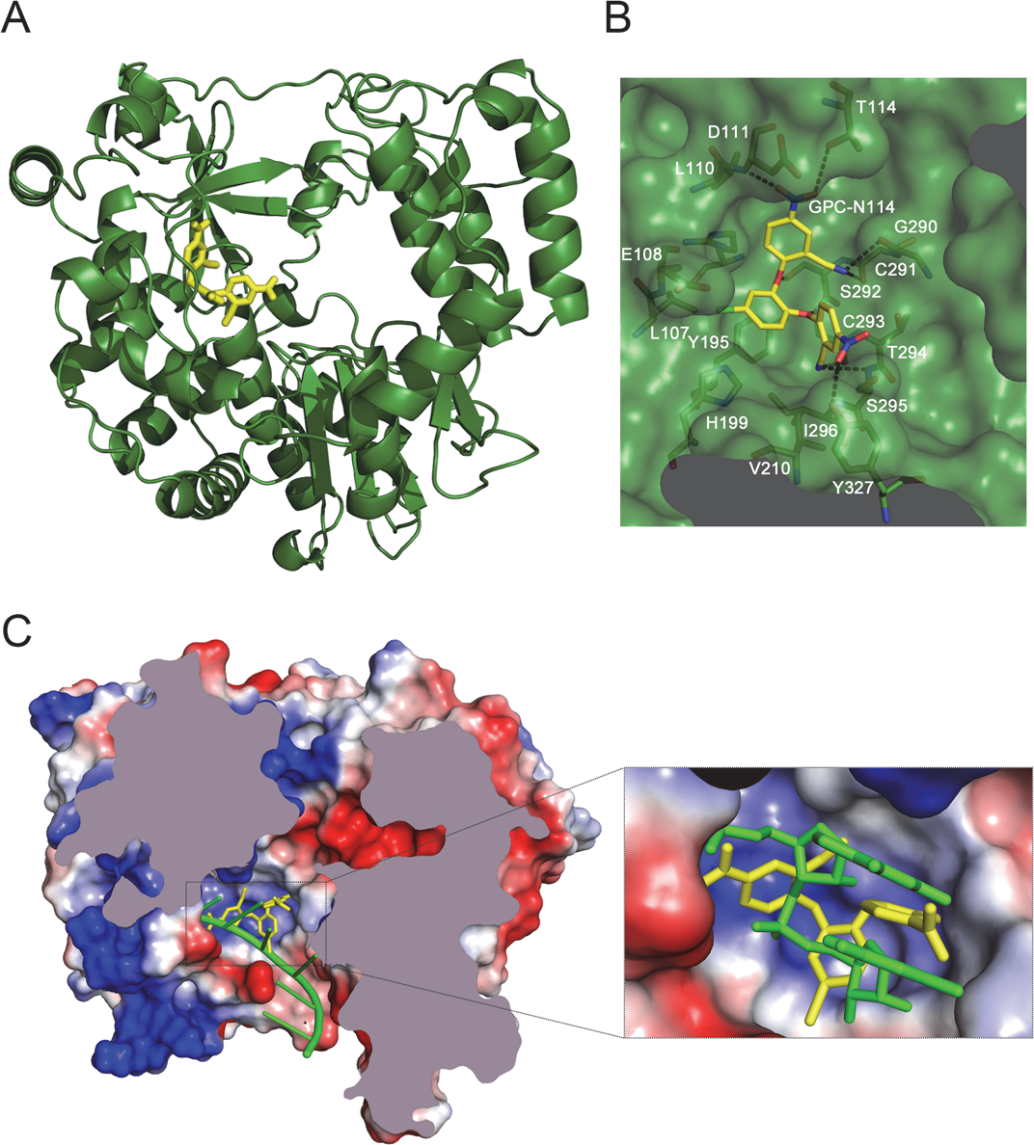
The binding site of GPC-N114 on CVB3 3D^pol^. (A) Ribbon diagram of the CVB3 3D^pol^ structure (green). The inhibitor bound to the bottom of the template channel is shown in stick representation in yellow. (B) Interaction network between GPC-N114 and its binding pocket of CVB3 3D^pol^ represented by surfaces. The polymerase residues in direct contact with the inhibitor are shown with carbon atoms in green and explicitly labeled. Hydrogen bonds are depicted as dashed lines. (C) Top down cross-sectional view of the molecular surface of CVB3 3D^pol^ with the electrostatic potential, represented in blue and red for positive and negative charges, respectively. The trajectory of the RNA template (green) is compared with the position of the GPC-N114 inhibitor (yellow). The inset box shows a close up of the position of the inhibitor that is coincident with that of the template acceptor nucleotide. The predicted position of the RNA template was determined using the FMDV 3D^pol^–RNA complex (PDB entry 1WNE) as a guide.

Co-crystallization of 3D^pol^ in complex with GPC-N114 was attempted to study the binding of the compound to picornavirus polymerases. Co-crystals of EMCV 3D^pol^ with the inhibitor were not obtained, but the structure of the complex of CVB3 3D^pol^ with GPC-N114 was obtained by X-ray crystallography at 2.9 Å resolution (**Table 2**). The analysis of the difference electron density maps revealed the presence of extra densities to position the inhibitor within a pocket located at the bottom of the template channel, mimicking the position of the template acceptor nucleotide, the nucleotide which will base-pair with the incoming nucleotide (**Fig. 3A-C and S6 Fig.**). The GPC-N114-binding pocket is in close proximity to the location of the mutated residues in resistant EMCV 3D^pol^ (compare **Fig. 2D** and **Fig. 3A**). The compound bound in a conformation that perfectly fitted the shape of the pocket formed by residues L107, E108, L110, D111, T114 of motif G, R188, Y195, H199 of motif F, T294, S295, I296 of motif B and Y327 of motif A (**Fig. 3A and 3B**). Most of these residues are conserved in picornaviral polymerases (**S7 Fig.**). Among all contacts observed, the stacking interaction between Y195 and the central chlorophenyl ring of GPC-N114 appeared to be an important determinant of binding. This tyrosine is strictly conserved in enterovirus polymerases but not conserved in FMDV and EMCV. The first 2-cyano-4-nitrophenyl ring was oriented towards the entrance of the template channel, interacting with residues of motif G and B of CVB3 3D^pol^, whereas the second 2-cyano-4-nitrophenyl ring appeared partially exposed to the solvent, occupying the position expected for the template acceptor base (**Fig. 3C**). The interacting residues appear to be in direct contact with the RNA templates in all picornaviral 3D^pol^–RNA complexes determined so far [4,12,30,31]. Structural comparisons between unbound and GPC-N114-bound CVB3 3D^pol^ revealed that the polymerase did not undergo any major conformational change upon binding of the compound. This preservation of the template cavity conformation extended to the interacting side chains, which maintained their native conformation upon GPC-N114 binding.

**Table 2:** Data collection and refinement statistics.

Data collection	CVB3 3D^pol^–GPC-N114	CVB3 3D^pol^–GPC-N143	EMCV 3D^pol^-M300V	EMCV 3D^pol^-I304V
Space Group	P4_3_2_1_2	P4_3_2_1_2	I4_1_22	C2
Cell dimensions				
a, b, c (Å)	74.74, 74.74, 289.1	74.62, 74.62, 288.43	122.6, 122.6, 194.5	232.3, 140.8, 171.8
α, β, γ (°)	90, 90, 90	90, 90, 90	90, 90, 90	90, 126, 90
Rmerge	8.1	7.2	8.1	13.7
I/σI	7.6	9.2	9.0	5.2
Completeness (%)	99.8	96.8	94.5	99.5
Multiplicity	3.6	3.6	2.8	2.9
**Refinement**				
Resolution (Å)	72.4–2.9	72.2–2.7	43.3–2.2	49.4–3.2
No. reflections (unique)	18099	21638	34387	69711
Rwork† / Rfree‡	21.1/ 22.9	20/24	22/26	21/25
No. Atoms				
- Protein	3746	3765	3711	22088
- Ligand	31	31	0	0
- GOL + ions	49	49	24	61
- Water	81	49	176	72
B-factors (Å ^2^ )				
- Protein	69.39	48.47	31.3	78.9
- Water + Ligands	65.51	59.69	47.3	59.55
R.m.s. deviations				
- Bond lengths (Å)	0.011	0.019	0.0042	0.0049
- Bond angles (°)	1.319	1.79	0.879	0.912
Ramachandran plot				
- Residues in preferred regions	370 (89.8%)	431 (83.49%)	450 (98.3%)	2586 (94.1%)
- Residues in allowed regions	42 (10.2%)	30 (6.51%)	8 (1.7%)	154 (5.6%)

† Rwork = ∑hkl ||Fobs(hkl)|—|Fcalc(hkl)|| / ∑hkl |Fobs(hkl)|, where Fobs and Fcalc are the structure factors, deduced from measured intensities and calculated from the model, respectively.

‡ Rfree = as for Rwork but for 5% of the total reflections chosen at random and omitted from refinement.

We also solved the X-ray structure of the complex of CVB3 3D^pol^ with a second inhibitor of the GPC series, GPC-N143, at 2.7 Å resolution (**S8A Fig. and Table 2**). This compound contains a central fluorophenyl ring instead of a chlorophenyl ring and displays similar antiviral activity as GPC-N114 (**S8A** and **S8B Fig.**). As expected, the GPC-N143 binding site is identical to that of GPC-N114, establishing almost equivalent interactions (**Fig. 3B**, **S6 Fig.** and **S8D Fig.**). Thus, the crystallographic data demonstrate that GPC-N114 and GPC-N143 specifically interact with CVB3 3D^pol^ at the template channel without having major effects on the structure of the enzyme.

### Model of EMCV 3D^pol^–GPC-N114 interactions and the structure of M300V and I304V mutants

**Fig 4:**
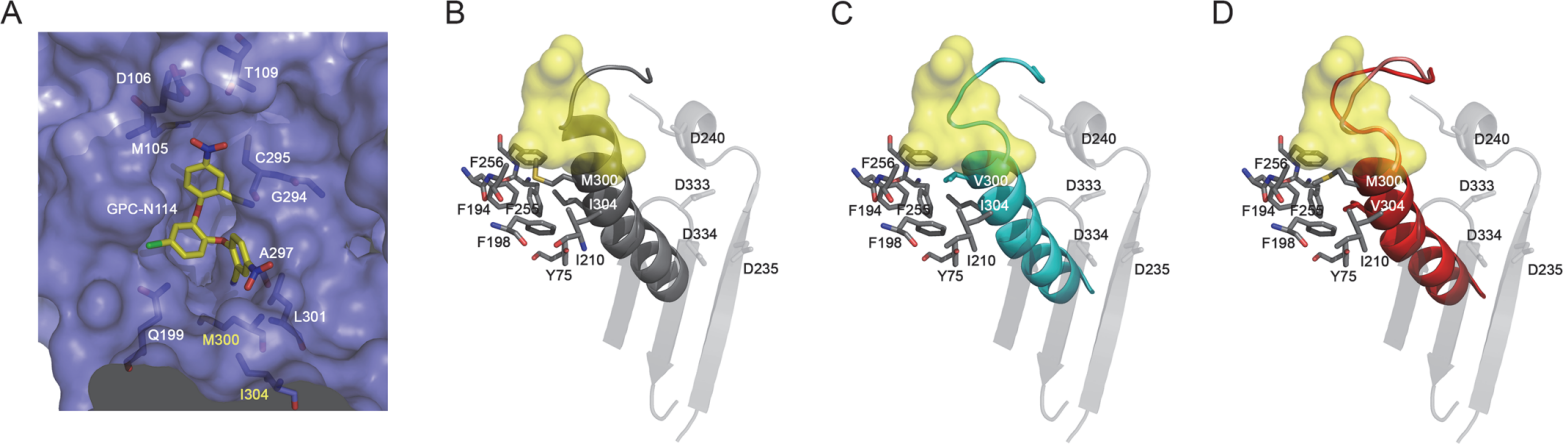
Effects of mutations M300V and I304V on the GPC-N114 binding site in EMCV 3D^pol^. (A) Surface representation of the putative GPC-N114 binding pocket in EMCV 3D^pol^ (slate blue) with the modeled inhibitor depicted in atom color (carbon atoms in yellow). (B-D) Environment of 300 and 304 residues on EMCV wt and mutants 3D^pol^. Motifs A and C are represented in transparent grey and motif B in solid color: (B) grey (wt), (C) cyan (M300V) and (D) red (I304V). Residues interacting with motif B α-helix are shown as sticks colored by atom. The GPC-N114 binding site on CVB3 3D^pol^ is represented as a yellow surface.

All attempts to obtain the structure of EMCV 3D^pol^–GPC-N114 complex were unsuccessful, however, the superposition of the CVB3 3D^pol^–GPC-N114 complex onto the unbound EMCV 3D^pol^ structures allowed us to define the putative inhibitor binding site in the EMCV enzyme (**Fig. 4A**). The overall shape of this site is similar in the two enzymes, but some important contacts are lost in EMCV (e.g., the stacking interaction between the side chain Y195 and the central ring of GPC-N114 is absent due to the substitution of this residue by alanine in EMCV). The model also shows that the major polymerase–inhibitor interactions are mediated by residues of the motif B loop (β10-α10) (**Fig. 4A and S7 Fig.**). The resistance mutations at EMCV 3D^pol^ residues 300 and 304 of helix α10 are close to the putative GPC-N114 binding site. However, only the long lateral chain of the EMCV wt residue M300 is able to establish a direct contact with the compound.

The apo structures of the EMCV M300V and I304V polymerase mutants were obtained using the same crystallization conditions as for the wt enzyme [29], yielding two different types of crystals for the mutants. The M300V mutant crystallized in the space group I4_1_22 form with one 3D^pol^ molecule in the asymmetric unit (a.u.) and diffracted to 2.2 Å resolution. The 3D^pol^ I304V mutant crystallized in the space group C2 with six polymerase molecules per a.u. and diffracted to 3.2 Å resolution. Data collection and refinement statistics are summarized in **Table 2**. Structural comparisons showed that the mutant polymerases were almost identical to the wt enzyme. The main differences were concentrated in helix α10, which contains the mutated residues, and in the preceding loop B. The side chains of residues 300 and 304 participate in a large hydrophobic cluster linking α10 with helices α2 (Y75), α6 (F194, A195, F199), α7 (I210) and α8 (L241, F255 and F256), included in the inner fingers. The substitutions of the methionine and isoleucine side chains by the shorter valine in the mutants imply a weakening of these hydrophobic interactions. This could result in an increased flexibility of α10, including the disruption of the last helical turn, and of the B-loop, which appears to have a key role in GPC-N114 binding in EMCV 3D^pol^ (**Fig. 4**). In addition, the substitution with valine may disrupt the interaction of residue 300 with the compound.

### GPC-N114 has a stronger inhibitory effect when added to 3D^pol^ before the RNA template-primer duplex

**Fig 5:**
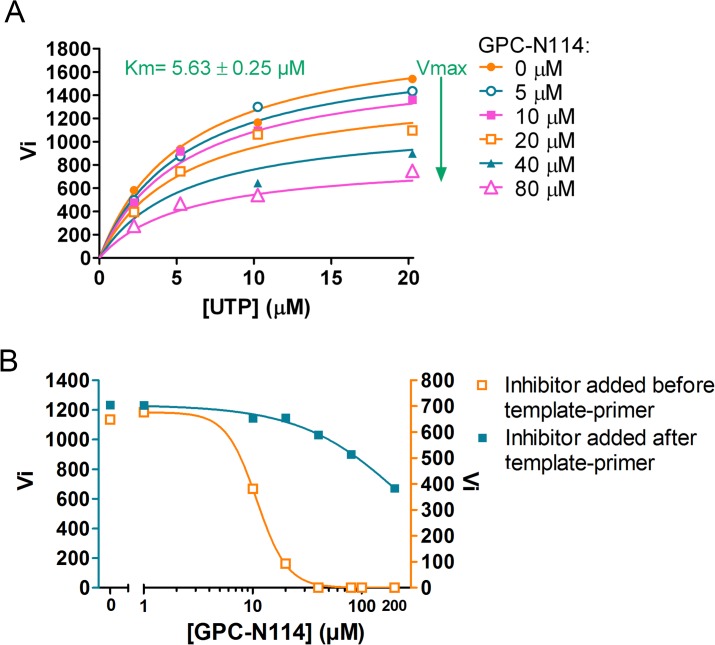
Characterization of the inhibitory mechanism of GPC-N114 on CVB3 3D^pol^. (A) GPC-N114 does not compete with UTP. The experiment was performed for CVB3 3D^pol^ as described in Fig. 2E for EMCV 3D^pol^. (B) GPC-N114 is more efficient when added to the reaction mix before the template-primer. Order-of-addition experiments were performed by measuring CVB3 3D^pol^ elongation activity when GPC-N114 was added to the reaction mix before or after poly(rA)/dT15. A typical of three independent experiments is presented.

Prompted by the identification of the binding site of GPC-N114 on CVB3 3D^pol^, we analyzed the effects of GPC-N114 on CVB3 3D^pol^ in more detail. Consistent with the data obtained for EMCV 3D^pol^, GPC-N114 inhibited CVB3 3D^pol^ elongation activity of the homopolymeric substrate poly(rA)/dT15 (**Fig. 5A**). Again inhibition was noncompetitive with respect to UTP. Since the binding sites of GPC-N114 and the template on CVB3 3D^pol^ overlapped, we aimed to explore whether GPC-N114 competes with the RNA template-primer duplex for binding to 3D^pol^. A possible method to test whether GPC-N114 precludes binding of the enzyme to RNA is by monitoring changes in fluorescence polarization signal [32] of a labeled symmetric heteropolymeric template-primer duplex (sym/sub-U) [33]. However, though GPC-N114 had an inhibitory effect in the elongation assays with the homopolymeric poly(rA)/dT15 template-primer (**Fig. 5A**), the compound failed to inhibit the incorporation of NTPs using the sym/sub-U template-primer, the latter being in contrast with what we observed in the elongation assays with the homopolymeric poly(rA)/dT15 template-primer (**S9 Fig.**). Therefore, an order-of addition assay was performed to compare the efficiency of GPC-N114 when added to the reaction mixture with 3D^pol^ before or after addition of poly(rA)/dT15. If GPC-N114 would affect productive binding of the template-primer to 3D^pol^, the inhibitor should display an improved potency when added to the reaction mix with 3D^pol^ before the template-primer as compared to after. When enzyme was pre-incubated with the inhibitor before adding the template-primer on average a 3-fold (n = 3) reduction in the Vmax was observed (**Fig. 5B**), which is consistent with the reported inactivation of picornavirus polymerases when they are incubated in the absence of nucleic acid [34]. Importantly, the IC_50_ value for GPC-N114 on CVB3 3D^pol^ was dramatically reduced (on average 14-fold, n = 3) when the compound was added to the reaction mix before the template-primer (**Fig. 5B**). Unfortunately, this assay is not able to discriminate between direct competition and the formation of a nonproductive ternary complex consisting of GPC-N114–template-primer–3D^pol^. Nevertheless, the observation that GPC-N114 has a stronger inhibitory effect on 3D^pol^ activity when added before the template-primer is compatible with the overlapping binding sites of the compound and the template-primer on 3D^pol^.

## Discussion

There is a great need for broad-range antiviral compounds to treat infections with enteroviruses, which includes important human pathogens such as poliovirus, coxsackievirus, enterovirus 71, and rhinovirus. Here, we report a novel small molecule, GPC-N114 that exerts broad-spectrum anti-enterovirus activity and also inhibits members of the genus *Cardiovirus*, such as EMCV. Analysis of its mechanism of action revealed that GPC-N114 inhibits virus replication at the stage of RNA replication. GPC-N114-resistant enterovirus variants could not be obtained, but compound-resistant EMCV variants were readily selected in the presence of suboptimal concentration of GPC-N114. These variants were found to carry mutations in the viral RdRP, 3D^pol^. Consistently, the *in vitro* elongation activity of CVB3 and EMCV 3D^pol^ was inhibited by GPC-N114, and the mutations identified in compound-resistant EMCV 3D^pol^ rendered this polymerase less susceptible to inhibition. GPC-N114 did not compete with incoming NTPs, but interfered with productive binding of the template-primer to 3D^pol^ in a primer elongation assay. This is in agreement with the crystallographic studies of the CVB3 3D^pol^–GPC-N114 complex which revealed that the binding site of the compound is located at the junction of the palm and the fingers domains, and overlaps partially with the binding site of the template.

A high barrier to resistance is a desirable feature for antiviral compounds since drug resistance represents a major problem in the treatment of viral infections. Notably, no GPC-N114-resistant enteroviruses (CVB3, PV1, E9) were obtained after several attempts and a large number of serial passages of these viruses in the presence of suboptimal concentrations of the compound. Attempts to generate compound-resistant CVB3 by introduction of the mutations that correspond to those identified in GPC-N114-resistant EMCV (i.e., 3D-I296V and 3D-M300V in CVB3), also did not result in a compound-resistant phenotype. The reason for the inability of enteroviruses to develop resistance against GPC-N114 remains to be established. A possible explanation is that mutations that would confer resistance to GPC-N114 also impair binding of the template-primer, thereby preventing replication. Although the exact reason remains to be determined, our data suggest that the compound-binding site in enterovirus 3D^pol^ lacks the conformational plasticity for resistance to develop, in contrast to most allosteric binding sites at the enzyme surface.

In contrast, EMCV 3D^pol^ is sufficiently plastic to allow for substitutions that result in compound-resistance. The amino acid changes are conservative with all three amino acids having similar properties. Based on the constructed model, only the long side chain of M300 (equivalent to I296 in CVB3 3D^pol^, see **Fig. 3B**), would be in direct contact with the compound. In contrast, I304 seems to be located relatively far away of the GPC-N114 binding cavity. Both M300 and I304 are part of conserved structural motif B, which not only binds the RNA template in the active site but also participates in the mechanism of RNA translocation through fine movements of a loop located at the N-terminus of the motif, the B-loop [30,35]. Structural data from picornavirus polymerases [30,35] as well as other RdRPs [36] suggest that the B-loop is highly dynamic and that its dynamics can be regulated by external effectors [37]. The structures of the EMCV 3D^pol^ M300V and I304V mutants suggest that these mutations increase the flexibility of this loop, thereby affecting the binding site of GPC-N114 and probably losing a direct interaction with the shorter V300 side chain whilst retaining the ability to interact with the template-primer.

EMCV 3D^pol^ and CVB3 3D^pol^ display a high structural similarity, but there is a major difference in the main interactions of these polymerases with GPC-N114 that could possibly underlie the difference in resistance emergence. In CVB3 3D^pol^, the main interaction with GPC-N114 is mediated by Y195. EMCV 3D^pol^, however, possesses an alanine at this position which forms a weaker interaction (**S10 Fig.**). Due to this weaker interaction, a flexibility increase in the compound-binding area induced by the B loop resistance mutations may result in a decreased binding of GPC-N114 to EMCV 3D^pol^. For CVB3 3D^pol^, a possible increase in flexibility induced by B loop mutations may not, or to a lesser extent, affect the binding of GPC-N114 due to its strong interaction with Y195.

GPC-N114 proved to be not a very robust compound in biochemical assays, for which the reason might be that it interferes with the binding of a by far larger molecule, the RNA template-primer. Thus, despite our efforts, it remains to be established whether GPC-N114 inhibits 3D^pol^ elongation activity by competing with the template-primer for binding to 3D^pol^ or whether binding of the compound results in the formation of an unproductive conformation of the polymerase-template complex. Nevertheless, both the crystallographic studies as well as the order-of-addition experiment strongly suggest that GPC-N114 affects binding of the template-primer by 3D^pol^. Compounds that interfere with binding of the template-primer have been described for polymerases of some viruses, including DENV and HIV [38,39], but not for any of the picornaviruses. The only known picornavirus polymerase inhibitors with a related mechanism of action are two compounds that target the incoming NTP binding site in the PV polymerase [15]. Clearly, the NTP binding site is close to, but distinct from, the GPC-N114 binding site. Hence, GPC-N114 is the first reported picornavirus polymerase inhibitor that binds the site of the template acceptor nucleotide.

Polymerase inhibitors have demonstrated their importance for the treatment of other viral infections such as those caused by HIV, herpesvirus, HBV and HCV. Unfortunately, it was not possible to evaluate the antiviral effect of GPC-N114 in an animal model, since—despite extensive attempts—we have not been able to formulate GPC-N114 (or GPC-N143) for administration. Notwithstanding this, the identification of a new druggable pocket in the polymerase through the characterization of the mechanism of action of GPC-N114 may be key in the (*in silico*) design of novel picornavirus polymerase NNIs.

## Materials and Methods

### Cells and reagents

Buffalo green monkey (BGM) kidney cells, HeLa R19 cells (obtained from G. Belov, University of Maryland and Virginia-Maryland Regional College of Veterinary Medicine, US), baby hamster kidney 21 (BHK-21) cells, and human rhabdomyosarcoma RD cells were grown at 37°C, 5% CO_2_ in Dulbecco’s Modified Eagle medium (DMEM) (Gibco) supplemented with 10% fetal bovine serum and antibiotics [28,40]. The Hela H cells, BGM cells, and Vero cells used for the multicycle CPE-reduction assays were maintained in Minimal Essential Medium supplemented with 10% FBS, 1% bicarbonate, and 1% L-glutamine [28].

The synthesis of GPC-N114 will be published elsewhere. Guanidine hydrochloride (GuHCl) and dipyridamole were purchased from Sigma Aldrich. Dipyridamole was dissolved in ethanol and GuHCl in water. The other compounds were dissolved in DMSO. GPC-N114 was used in a concentration of 10 μM unless stated otherwise.

### Viruses and infections

Coxsackievirus B3 (strain Nancy) was obtained by transfection of RNA transcripts of the full-length infectious clone p53CB3/T7 linearized with SalI into BGM cells [41]. EMCV, strain mengovirus, was obtained in a similar manner by transfection of RNA derived from cDNA clone pM16.1 into BHK-21 cells [42]. EV68, EV70, EV71 (BrCr), E11 (Gregory), CVA16 (G-10) and CVA21 (Coe) were obtained from the National Institute for Public Health and Environment (RIVM, the Netherlands). Poliovirus 1–3 Sabin reference strains were obtained from dr. J Martin (NIBSC, UK). Human rhinoviruses 2 and 14 were supplied by Joachim Seipelt (Medical University of Vienna, Austria). ERAV (NM11/67) was a gift from David Rowlands and Toby Tuthill (University of Leeds, UK). Saffold virus (type 3) was described previously [43]. The FMDV strains O_1_ Manisa and A_22_ Iraq 24/64 were used [44].

For virus infections, virus was added to subconfluent cell layers and allowed to adsorb for 30 minutes, after which virus was removed and fresh (drug-containing) medium was added to the cells. At the indicated times p.i. the cells were freeze-thawed three times to release intracellular virus particles. Virus titers were determined by endpoint titration according to the method of Reed and Muench and expressed as 50% cell culture infective doses (CCID50)/ml [45].

### Multicycle CPE-reduction assays

The antiviral activity of compounds was determined as published previously [11,44]. In short, the medium was removed from cells grown in 96-well plates and a serial dilution of compound in medium with 2% FCS was added after which the cells were infected with virus at the lowest MOI sufficient for full CPE development in infected, untreated cultures after three days of incubation at 37°C. To quantify the level of CPE by MTS assay, the medium was then replaced with AQ_ueous_ One Solution Cell Proliferation Reagent (Promega) diluted in medium and incubated at 37°C for 1–2 h. Subsequently, absorbance at 498 nm was measured. The measured values were used to calculate the cell viability ranging from 0% (full CPE with infected, untreated cultures) to 100% (uninfected, untreated cultures). The 50% effective concentration (EC50) was defined as the concentration of compound that resulted in a 50% reduction in virus-induced CPE and was calculated using logarithmic interpolation.

### Subgenomic replicon assay

The subgenomic replicons of CVB3 (pRib-LUC-CB3/T7) and EMCV (pEMCV-FLuc) encode the cDNA clones of the corresponding virus except that the P1 area encoding the capsid proteins has been (partially) replaced by the code for firefly luciferase [24,46]. The plasmids were linearized with MluI or BamHI, respectively. RNA transcribed from the linearized plasmids was used to transfect BGM cells plated in 24-well plates using the DEAE-dextran method as described previously [24]. After transfection, cells were treated with DMSO or compound and incubated at 37°C. At 2, 4, 6, and 8 h post-transfection, cells were washed with PBS, lysed and luciferase levels were determined with the Luciferase Assay System (Promega) according to the manufacturer’s instructions.

A similar procedure was followed for the PV1 subgenomic replicon assay pXpA-RenR-PV except that luciferase activity was measured with the *Renilla* Luciferase Assay System (Promega), since this construct encodes the *Renilla* luciferase [47].

### Metabolic labeling

For metabolic labeling of proteins, BGM cells were grown in 24-well plates to subconfluence and infected with CVB3 at an MOI of 50. At 5 h post-infection, the medium was replaced with 300 μl of methionine-free medium for 30 min. Subsequently, the cultures were pulse-labeled in methionine-free medium supplemented with [^35^S]Met in the absence or presence of 50 μM GPC-N114 for 30 min. At 6 h post-infection, cells were lysed with lysis buffer [50 mM Tris (pH 7.4), 150 mM NaCl, 1 mM EDTA, 1% Nonidet P-40, 0.05% SDS]. Laemmli sample buffer was added to the lysates, boiled for 5 min and analyzed on a 10% polyacrylamide gel containing SDS. The gels were fixed in 30% methanol-10% acetic acid, rinsed in DMSO, fluorographed with 20% 2,5-diphenyloxazole in DMSO, dried, and exposed to Kodak XAR film.

### Generation of GPC-N114-resistant virus

To obtain drug-resistant virus, three independent virus pools of CVB3 and EMCV were serially passaged in the presence of a concentration series of GPC-N114 on subconfluent to confluent Vero cultures in 96-well plates. The amount of virus used for infection was selected so that full CPE was visible after 3 days of incubation in the absence of compound. The concentration series encompassed 8 different concentrations in 2-fold dilutions starting from 25–100 μM. After 3 to 4 days, the amount of CPE was assessed and supernatant collected from the culture with the highest concentration of compound that still exhibited full CPE was used to perform the next round of infection. This procedure was repeated 32 times (CVB3) or until there was a clear increase in the concentration required for inhibition of CPE (EMCV) compared to wt virus that was tested in parallel in each passage. Viral RNA was isolated from the resistant virus pools using the GenElute Mammalian Total RNA Miniprep Kit (Sigma Aldrich) and used for sequencing of the parts of the genome that encode the nonstructural proteins. The PCR product containing the mutations found in the virus pools generated in this procedure was digested with PinAI and NruI and cloned into pM16.1 to replace the corresponding wt sequence. The resulting infectious clones pM16.1-3D-M300V and pM16.1-3D-I304V were sequenced to confirm the presence of the mutations.

The analogous mutations in CVB3 (I296V and M300V) were introduced into the infectious cDNA p53CB3/T7CVB3 by site-directed mutagenesis. The mutant clones were sequenced to confirm the presence of the mutations. The construction of pRibCB3/T7 with the S299T mutation was described previously [48].

Recombinant EMCV and CVB3 was generated as described above and the presence of the mutations was again confirmed by sequence analysis.

### Protein expression and purification for biochemical activity assays

Recombinant CVB3 3D^pol^ and the RdRP domain of DENV NS5 (NS5-RdRP) were obtained as previously described [49,50]. For expression and purification of EMCV 3D^pol^, the coding sequence of 3D^pol^ (polyprotein domain from amino acid position 1834 to 2293) was amplified on EMCV cDNAs (pM16.1 wt, 3D-M300V, and 3D-I304V) and cloned into pETG20A (kindly provided by Dr. Arie Geerlof) by Gateway recombination (Life Technologies), downstream a cleavable Hexahistidine-Thioredoxin tag using a two-step PCR protocol. The protein was expressed in E. coli Rosetta (DE3) pLysS strain (Novagen) at 17°C in Tur*best Broth (Arcadia Biotech). The purification of the protein and the tag removal were performed in non-denaturing conditions as previously described [51]. The final size exclusion chromatography step was performed in 10 mM Hepes, 300 mM NaCl, 2mM dithiotreitol, pH 7.5.

### Polymerase activity assays

The conditions for the polymerase elongation activity assays were adapted from conditions reported previously [10,50]. EMCV 3D^pol^ filter-binding polymerase activity assays were conducted in a mix of 50 μl containing 50 mM MOPS pH 7.0, 10 mM KCl, 4 mM MgCl_2_, 9% glycerol, 2 μM dT15 (Invitrogen) annealed to 350 nM poly(rA) (GE Healthcare, average size 519 nt) (i.e., a molar ratio dT15:poly(rA) of 5.7:1), 1 μM EMCV 3D^pol^, 50/100/200 or 500 μM UTP and 2 μCi [^3^H]UTP (GE Healthcare, 35.5 Ci/mmol). The reaction mix excluding UTP was incubated with either DMSO or GPC-N114 with a constant concentration of 5% DMSO for 2 minutes after which the reaction was started by addition of labeled and unlabeled UTP. Samples of 10 μl were taken after 0, 10, 20 and 30 min of incubation at 30°C and added to 50 μl of 100 mM EDTA in 96-well sample plates to stop the reaction. Mixtures were then transferred onto glass fiber filter mats with DEAE active groups (DEAE filtermat, Wallac) using a Filtermat Harvester (Packard Instruments). Filtermats were washed three times with 0.3 M ammonium formate pH 8.0, twice with water, and once with ethanol after which they were dried and transferred into sample bags. Liquid scintillation fluid was added to the sample bags and incorporation of radiolabeled UTP was measured in counts per minute using a Wallac MicroBeta TriLux Liquid Scintillation Counter.

For the DENV NS5 elongation assay the reaction mixture contained 50 mM HEPES pH 8.0, 10 mM KCl, 10 mM DTT, 5 mM MgCl_2_, 2 μM dT15 annealed to 350 nM poly(rA), 0.4 μM DENV NS5-RdRP, 20 μM UTP and 2 μCi [^3^H]UTP. 10 μl samples were taken after 0, 30, 60, and 90 minutes. Subsequently, the same procedure was followed as for EMCV 3D^pol^.

The CVB3 3D^pol^ reaction mixture contained 50 mM Tris pH 7.0, 10 mM KCl, 0.8 mM MgCl_2_, 9% glycerol, 2 μM dT15 annealed to 350 nM poly(rA), 20 nM CVB3 3D^pol^, UTP (2, 5, 10 or 20 μM) and 0.5 μCi [^3^H]UTP. 10 μl samples were taken after 0, 5, 10, and 15 minutes. For the order-of-addition experiment with CVB3 3D^pol^, a reaction premix was assembled as above but without poly(rA)/dT15, GPC-N114 and UTP. To this premix either a range of concentrations of GPC-N114 or 80 nM dT15 annealed to 14 nM poly(rA) were added. After an incubation of 1 min at RT, the other component was added to the mix followed by an incubation of several minutes at 30°C. The reaction was started by addition of UTP, and samples were taken after 0, 3, 6, and 9 minutes.

The data were fitted using non-linear regression using Graphpad Prism 5.0.3. Ki values averaged from at least three independent experiments were statistically compared between EMCV 3D^pol^ wt and the 3D^pol^ mutants using a Student’s *t*-test.

### Protein expression and purification for crystallography

Recombinant CVB3 3D^pol^ was over-expressed essentially as previously described [49], except that the expression was carried out at 20°C. Stored pellets were resuspended in a cold lysis buffer containing 50 mM Tris HCl pH 9.0, 500 mM NaCl, 10 mM imidazole, 0.1% (v/v) Triton X-100, 5% glycerol, 1 mg/ml lysozyme and 10 μg/ml DNase, and cells were lysed by sonication. Cellular debris was pelleted by centrifugation and the soluble fraction was filtrated and loaded into a 5 ml HisTrap HP column (GE Healthcare). The protein was eluted using an imidazole gradient (10–500 mM) in 50 mM Tris pH 8.0 and 300 mM NaCl. Protein fractions were dialyzed against 20 mM Tris pH 9.0, 600 mM NaCl, 15% glycerol, 0.5 mM TCEP and further purified by size-exclusion chromatography on a Superdex 200 16/60 column (GE Healthcare). Purified protein was concentrated to 5 mg/ml in the same buffer, using a Vivaspin concentrator (10.000 MWCO PES, Sartorius Stedim Biotech). The quality of the protein obtained was checked by SDS-PAGE.

EMCV 3D^pol^ wt and mutants were expressed and purified as previously reported for EMCV 3D^pol^ wt [29]. Briefly, using the constructs described above, the polymerases were overexpressed in E. coli, and EMCV 3D^pol^ was purified by means of Ni-affinity (HisTrap HP, GE Healthcare). After that, the His-tag was removed by TEV digestion, and size exclusion chromatography (Superdex 200 16/60, GE Healthcare).

### Crystallization and data collection

Crystals of isolated CVB3 3D^pol^ were obtained by the sitting-drop vapor-diffusion method at 16°C in 96-well plates (MRC, Swissci AG) using a Cartesian automated drop dispenser to mix 250 nl of 5 mg/ml protein solution with an equal volume of reservoir solution, containing 50 mM Tris pH 7.5, 24.5% (w/v) glycerol, 1.29 M ammonium sulfate. Highly reproducible crystals of 25x25x25 μm in size appeared between 2–3 days. Protein/inhibitor complexes were prepared by soaking CVB3 3D^pol^ crystals in the inhibitor solution for 72h. The inhibitor solutions contained 100 μM GPC-N114 or GPC-N143 in 2.5% DMSO, 0.65 M ammonium sulfate, 12.5% glycerol and 25 mM Tris pH 7.5. Crystals were then transferred to a cryo-protecting solution, containing 30% glycerol in the respective crystallization buffer prior to cooling by immersion in liquid nitrogen. The two datasets were collected at 100K by using synchrotron radiation at the European Synchrotron Radiation Facility (ESRF, Grenoble) on a Pilatus 6M S/N 60–104 detector at beamline ID 29, λ = 0.977Å. Diffraction images were processed with iMOSFLM [52] and internally scaled with SCALA (CCP4i [53]), achieving resolutions of 2.9Å and 2.7Å for the CVB3 3D^pol^–GPC-N114 and CVB3 3D^pol^–GPC-N143 crystals, respectively. Data collection statistics are given in Table 2. Coordinates have been deposited at the PDB with accession codes 4Y2A and 4Y34 for the complexes CVB3 3D^pol^–GPC-N114 and CVB3 3D^pol^–GPC-N143, respectively.

Crystals of EMCV 3D^pol^ wt and M300V and I304V, mutants were obtained as previously described [29]. The data sets were collected at the ALBA-CELLS synchrotron light facility (Barcelona) on a Pilatus 6M Dectris detector at beamline XALOC λ = 0.977Å. Diffraction images were processed with iMOSFLM and SCALA, achieving resolutions of 2.2Å and 3.2Å for the M300V and I304V respectively (Table 2). Coordinates have been deposited at the PDB with accession codes 4Y2C and 4Y3C for the structures of EMCV 3D^pol^-M300V and EMCV 3D^pol^-I304V, respectively.

### Structure determination and refinement

The initial electron density maps of the CVB3 3D^pol^–GPC-N114 and CVB3 3D^pol^–GPC-N143 complexes were obtained after rigid-body fitting of the coordinates of the 3D^pol^ apoenzymes (PDB id. 3DDK) to the new unit cells (Table 2), using the program Refmac5 [54,55]. In both structures, the weighted 2|Fo|-|Fc| and |Fo|-|Fc| difference map showed the presence of extra densities that permitted the initial positioning of the inhibitor molecules. Inhibitor structure files were generated using “Online SMILES Translator and Structure File Generator” (http://cactus.nci.nih.gov/translate/) and its respective CIF dictionaries were created by Grade [56]. Several cycles of automatic refinement, performed with Refmac5, were alternated with manual model rebuilding using Coot [57]. The refinement statistics are summarized in Table 2.

The first electron density maps for both EMCV 3D^pol^ mutants were obtained after rigid-body fitting of the EMCV 3D^pol^ wt coordinates to the new unit cells. The PDB id 4NYZ, of space group I4_1_22, was used as a model for the M300V structure and coordinates 4NZ0, of space group C2, were used as a model for the I304V mutant. Noncrystallographic symmetry (ncs) restraints were initially applied to the six independent molecules present in the asymmetric unit of the C2 crystals using Refmac5. However, the ncs restrained-refinement result was unstable because the presence of different conformations in many polymerase regions, especially in the surface-exposed loops, was affected by crystal packing interactions. Therefore, the variable regions were manually rebuilt using Coot, and the final cycles of automatic refinement with Refmac 5, were performed in absence of ncs restraints. The refinement statistics are given in Table 2.

## Supporting Information

S1 Fig Effect of GPC-N114 on cell viability and picornavirus replication. (A) GPC-N114 does not affect cell viability. BGM cells were treated with GPC-N114 for 8 h after which cell viability was determined with an MTS assay. Cell viability is expressed as percentage of DMSO-treated cells. Experiments were performed in triplicate and mean values ± SD are depicted. (B) Antiviral activity of GPC-N114 against picornaviruses. BGM cells were infected with the indicated viruses at an MOI of 0.5. GPC-N114 (10 μM) was added immediately after infection. At 0, 8 and 24 h p.i. virus titers were determined by endpoint titration. Experiments were done in triplicate and mean values ± SD are shown.
(TIF)

S2 Fig GPC-N114 does not affect polyprotein processing. BGM cells were infected with CVB3 at MOI 50. At 5 h p.i. cells were starved for methionine for 30 min after which produced proteins were labeled with [^35^S]Met in the presence of DMSO or 50 μM GPC-N114 for another 30 min. Subsequently, proteins were analyzed by SDS-PAGE.
(TIF)

S3 Fig Replication of PV1 is completely inhibited by GPC-N114. The experiment was performed as described in Fig. 1F.
(TIF)

S4 Fig Mutations I296V, M300V, and S299T in 3D^pol^ do not confer resistance to CVB3. (A) BGM cells were infected with CVB3 wt or mutants at an MOI of 0.5 for 30 min. Subsequently, the inoculum was replaced with medium containing DMSO, GPC-N114, or guanidine hydrochloride (GuHCl). Virus titers were determined by endpoint titration after 8 h. Experiments were performed in triplicate and mean values ± SD are depicted. (B) Dose-response curves of multicycle CPE-reduction assays with CVB3 wt and CVB3 3D-S299T on BGM cells. CPE was quantified by MTS assay at 3 d p.i. and is expressed as percentage of uninfected, untreated controls.
(TIF)

S5 Fig GPC-N114 has no effect on DENV NS5 RdRP elongation activity. DENV NS5 RdRP elongation activity in the presence of a range of concentrations of GPC-N114 was determined by measuring incorporation of [^3^H]UTP using poly(rA)/dT15 as template-primer. The activity observed with DMSO was set at 100%. Experiments were performed in triplicate and values shown are mean ± SD.
(TIF)

S6 Fig Stereoview of the Fo-Fc omit map (contoured at 3.0 σ) around the inhibitor pocket for the CVB3 3D^pol^–GPC-N114 complex. The polymerase residues in direct contact with the inhibitor are shown with carbon atoms in green and explicitly labeled. Hydrogen bonds are depicted as dashed lines.
(TIF)

S7 Fig Sequence alignment of picornavirus RdRPs. The strictly conserved residues are in red blocks and similar residues in blue boxes. The residues interacting with GPC-N114 are marked by green (CVB3) and blue squares (EMCV).
(TIF)

S8 Fig GPC-N143. (A) Antiviral activity of GPC-N143 against CVB3 and EMCV. The experiment was performed as in Fig. 1C. Experiments were performed in triplicate and mean values ± SD are depicted. (B) GPC-N143 does not affect cell viability. The experiment was performed as in S1A Fig. Experiments were performed in triplicate and mean values ± SD are depicted. (C) Structural formula of GPC-N143. (D) Stereoview of the Fo-Fc omit map (contoured at 3.0 **σ**) around the inhibitor pocket for the CVB3 3D^pol^–GPC-N143 complex. The inhibitor contacting residues in the polymerase binding pocket are indicated.
(TIF)

S9 Fig Sym/sub-U assay. (A) The sequence of the sym/sub template primer duplex. (B-C) GPC-N114 has no effect on NTP incorporation in the sym/sub-U assay. NTPs (500 μM) were incubated in a buffer containing 50 mM Tris pH 7.0, 10 mM KCl, and 0.8 mM MgCl_2_ for 5 min. GPC-N114 or DMSO were added and the mix was incubated for another minute, followed by addition of 1 μM CVB3 3D^pol^. After a two-minute incubation, the reaction was initiated with [^32^P]-labeled sym/sub-u (1 μM) and quenched at 30, 60, 90, 120, 180 and 300s after the initiation. Reaction products were analyzed by electrophoresis on a denaturing polyacrylamide gel (C). The quantification of the incorporation of NTPs is depicted in (B).
(TIF)

S10 Fig Effect of Y195 on the binding of GPC-N114. Cartoon representation of the GPC-N114-binding pocket in CVB3 3D^pol^ (forest green) (A) and the putative inhibitor pocket in EMCV (slate blue) (B). Polymerase side chains are represented in sticks only for: i) Y195 in CVB3 3D^pol^, making crucial interactions with the compound, and its equivalent (A195) in EMCV 3D^pol^, ii) the EMCV residues M300 and I304 that are mutated in GPC-N114-resistant mutants and the equivalent amino acids in CVB3 3D^pol^ (I296 and M300). The inhibitor is represented in atom-type sticks with carbon atoms in yellow.
(TIF)
