# Supplementary figures and images for: The RNA Template Channel of the RNA-Dependent RNA Polymerase as a Target for Development of Antiviral Therapy of Multiple Genera within a Virus Family
Source: PLoS Pathog. 2015 Mar 23;11(3):e1004733. doi: 10.1371/journal.ppat.1004733 (PMC4370873; doi:10.1371/journal.ppat.1004733)

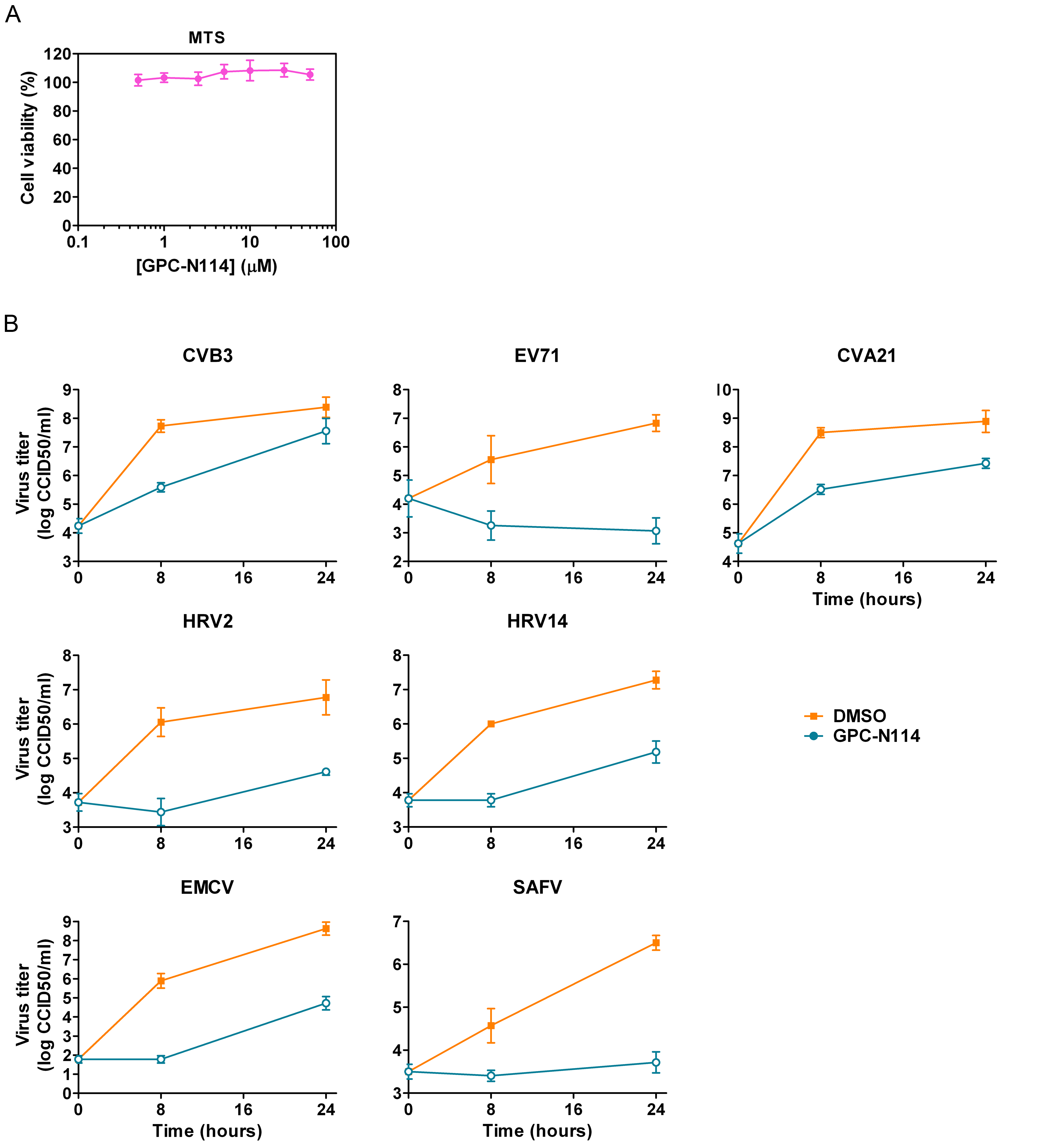

Supplement: S1 Fig — (A) GPC-N114 does not affect cell viability. BGM cells were treated with GPC-N114 for 8 h after which cell viability was determined with an MTS assay. Cell viability is expressed as percentage of DMSO-treated cells. Experiments were performed in triplicate and mean values ± SD are depicted. (B) Antiviral activity of GPC-N114 against picornaviruses. BGM cells were infected with the indicated viruses at an MOI of 0.5. GPC-N114 (10 μM) was added immediately after infection. At 0, 8 and 24 h p.i. virus titers were determined by endpoint titration. Experiments were done in triplicate and mean values ± SD are shown. (TIF) [file ppat.1004733.s001.tif]

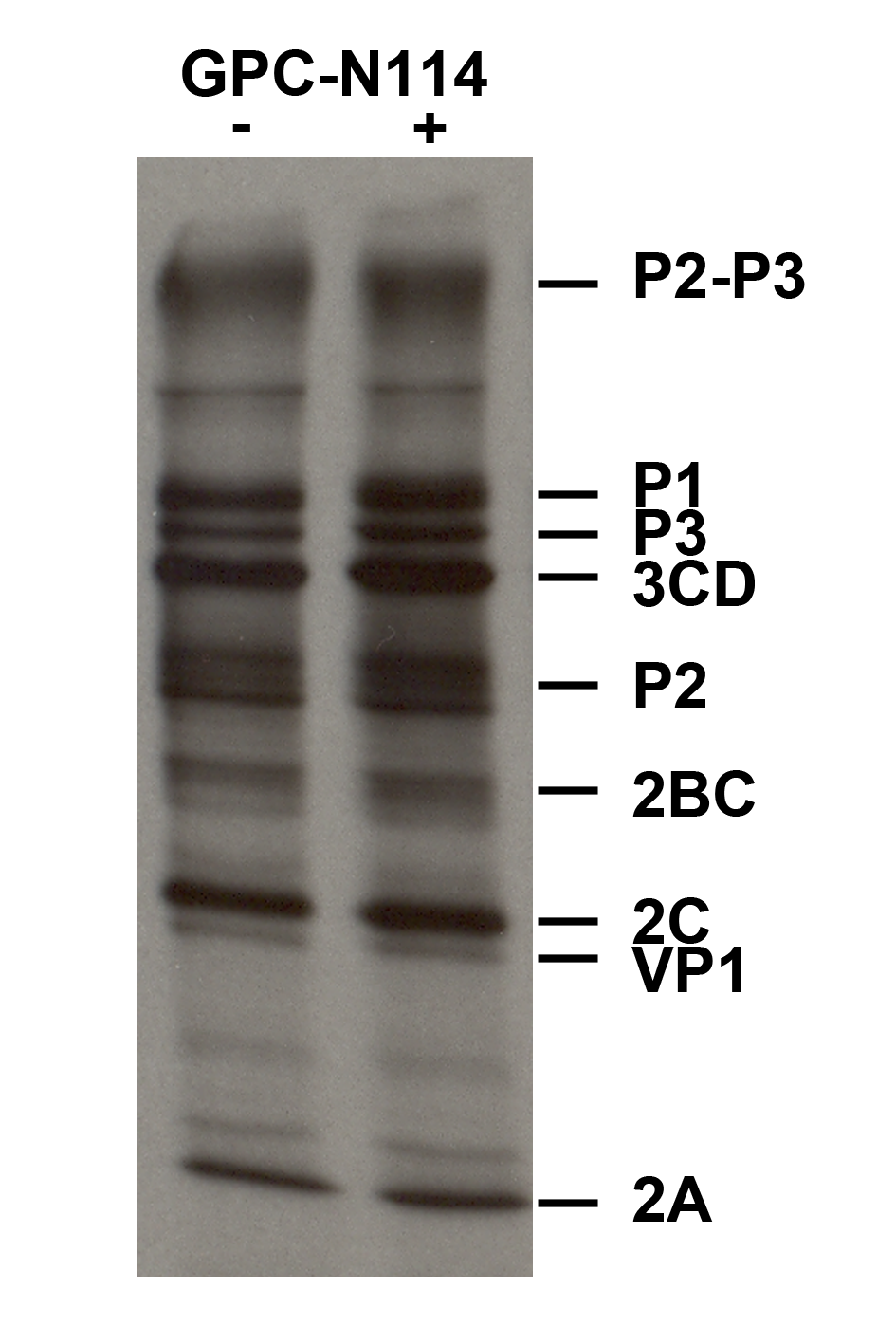

Supplement: S2 Fig — BGM cells were infected with CVB3 at MOI 50. At 5 h p.i. cells were starved for methionine for 30 min after which produced proteins were labeled with [35S]Met in the presence of DMSO or 50 μM GPC-N114 for another 30 min. Subsequently, proteins were analyzed by SDS-PAGE. (TIF) [file ppat.1004733.s002.tif]

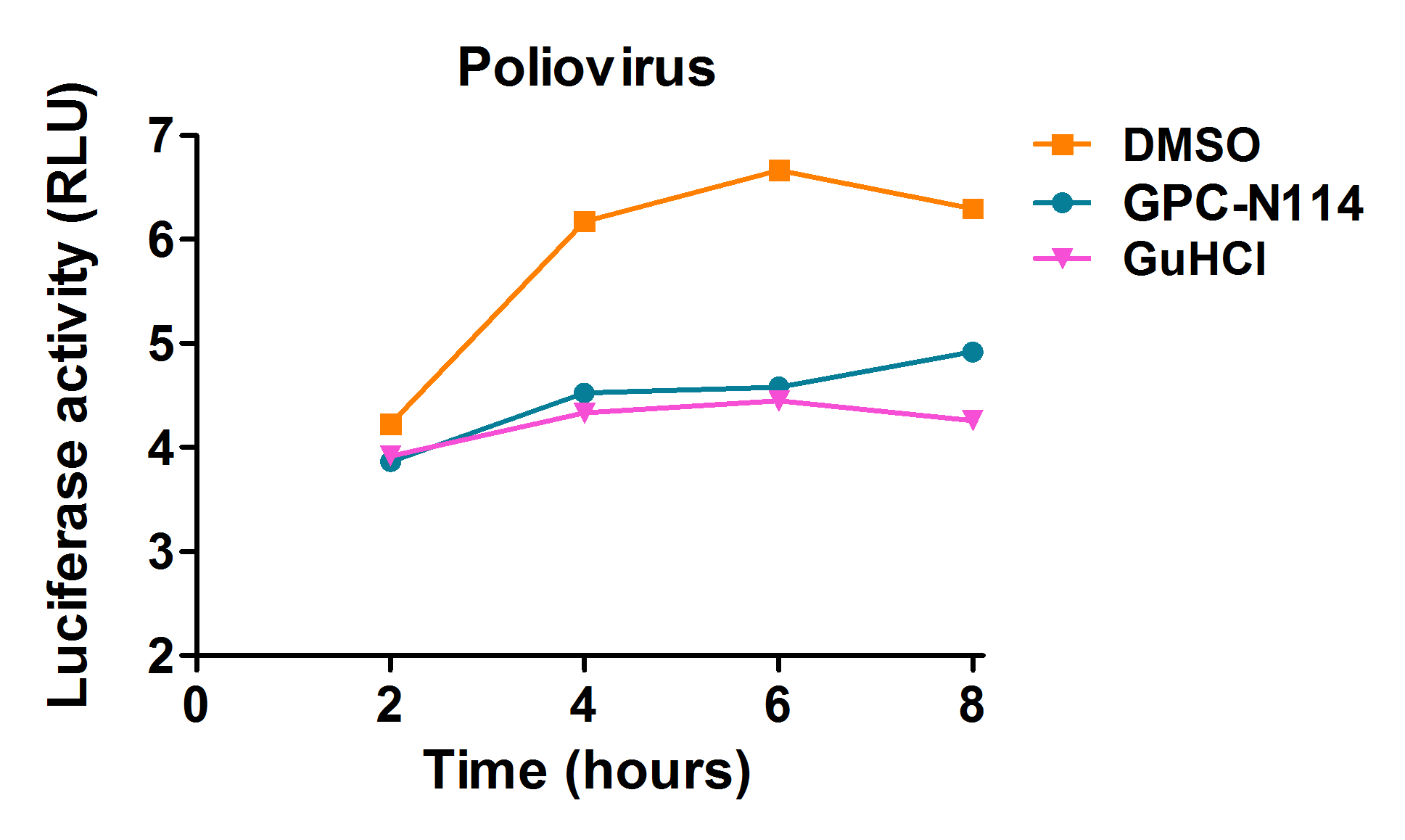

Supplement: S3 Fig — The experiment was performed as described in Fig. 1F. (TIF) [file ppat.1004733.s003.tif]

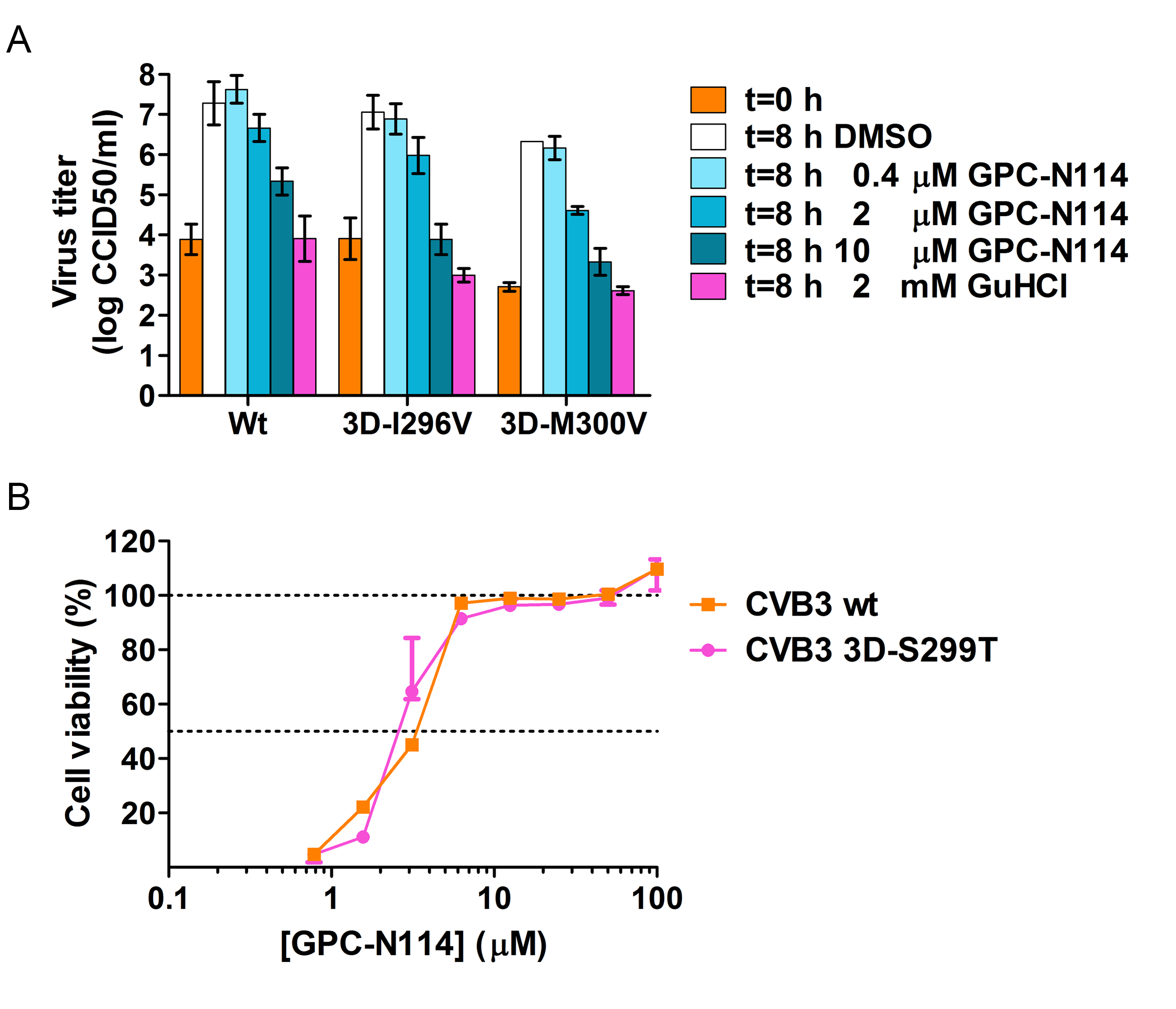

Supplement: S4 Fig — (A) BGM cells were infected with CVB3 wt or mutants at an MOI of 0.5 for 30 min. Subsequently, the inoculum was replaced with medium containing DMSO, GPC-N114, or guanidine hydrochloride (GuHCl). Virus titers were determined by endpoint titration after 8 h. Experiments were performed in triplicate and mean values ± SD are depicted. (B) Dose-response curves of multicycle CPE-reduction assays with CVB3 wt and CVB3 3D-S299T on BGM cells. CPE was quantified by MTS assay at 3 d p.i. and is expressed as percentage of uninfected, untreated controls. (TIF) [file ppat.1004733.s004.tif]

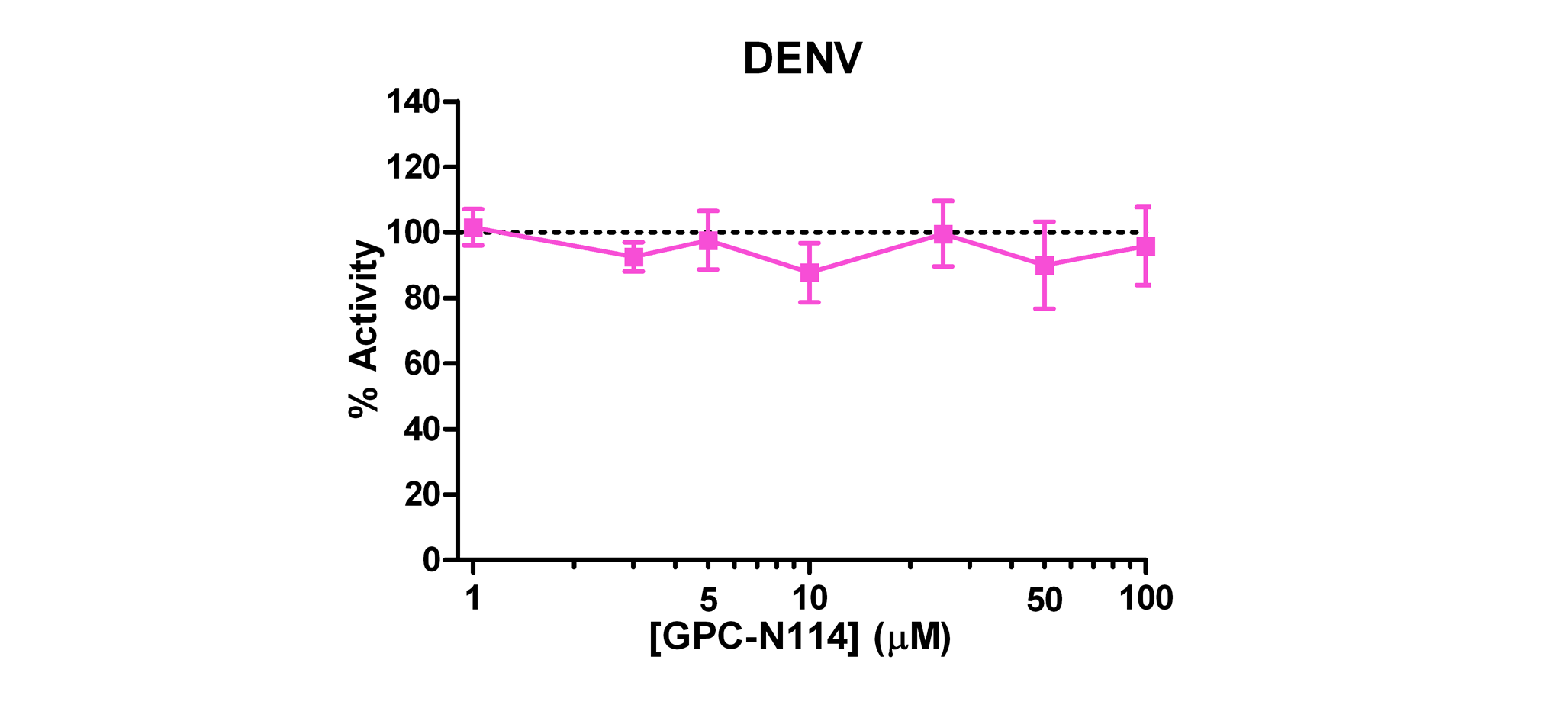

Supplement: S5 Fig — DENV NS5 RdRP elongation activity in the presence of a range of concentrations of GPC-N114 was determined by measuring incorporation of [3H]UTP using poly(rA)/dT15 as template-primer. The activity observed with DMSO was set at 100%. Experiments were performed in triplicate and values shown are mean ± SD. (TIF) [file ppat.1004733.s005.tif]

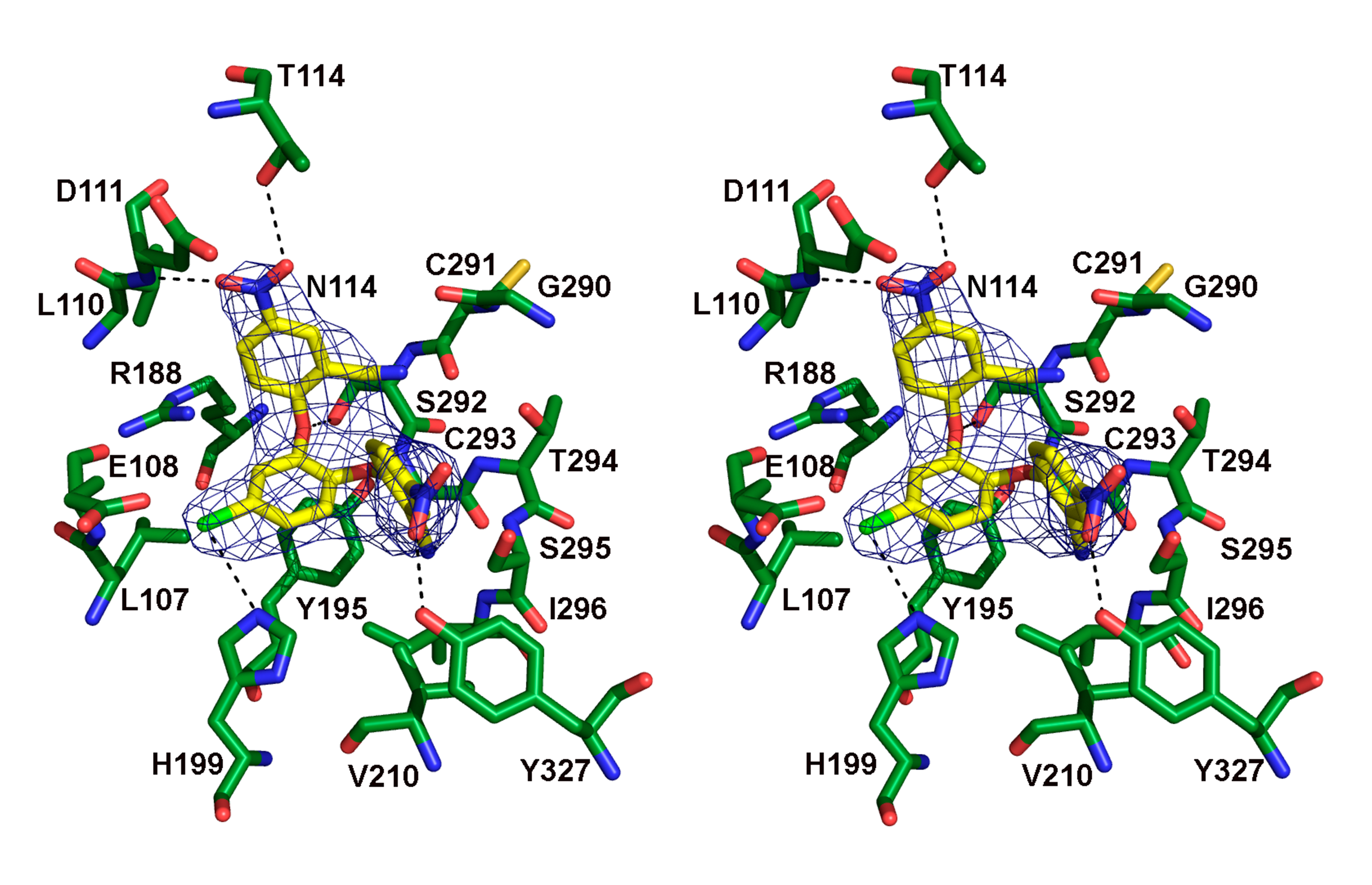

Supplement: S6 Fig — The polymerase residues in direct contact with the inhibitor are shown with carbon atoms in green and explicitly labeled. Hydrogen bonds are depicted as dashed lines. (TIF) [file ppat.1004733.s006.tif]

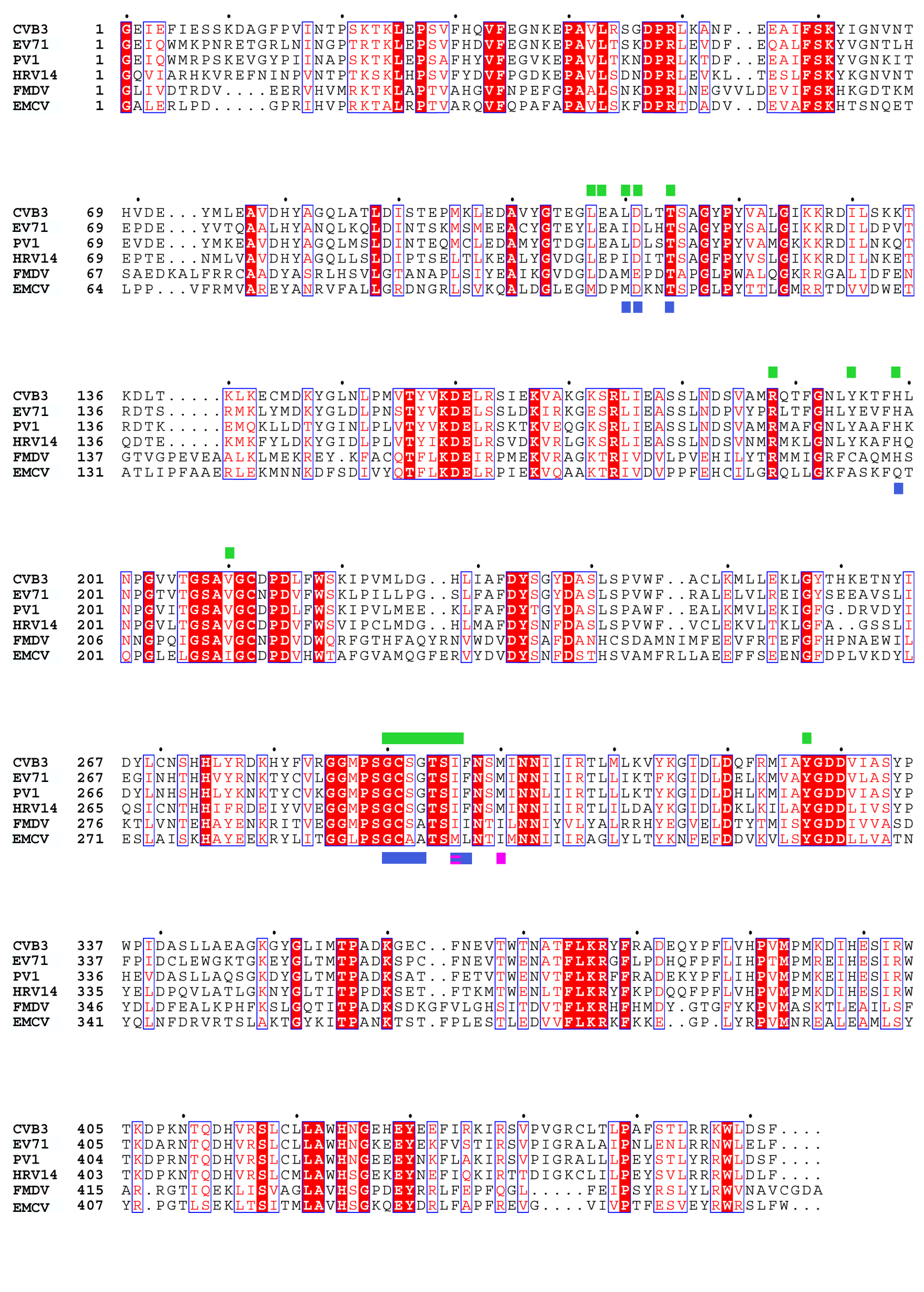

Supplement: S7 Fig — The strictly conserved residues are in red blocks and similar residues in blue boxes. The residues interacting with GPC-N114 are marked by green (CVB3) and blue squares (EMCV). (TIF) [file ppat.1004733.s007.tif]

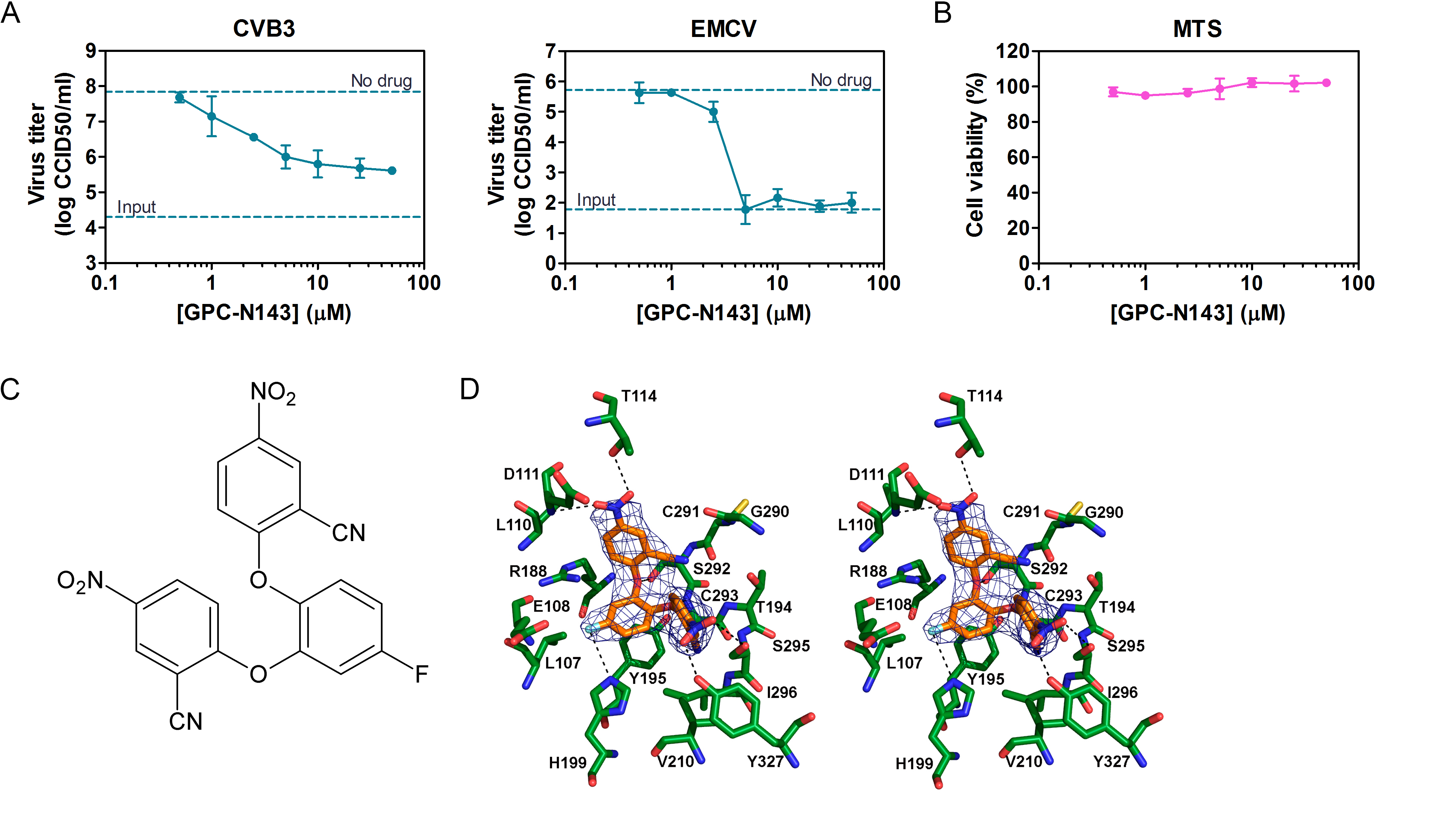

Supplement: S8 Fig — (A) Antiviral activity of GPC-N143 against CVB3 and EMCV. The experiment was performed as in Fig. 1C. Experiments were performed in triplicate and mean values ± SD are depicted. (B) GPC-N143 does not affect cell viability. The experiment was performed as in S1A Fig. Experiments were performed in triplicate and mean values ± SD are depicted. (C) Structural formula of GPC-N143. (D) Stereoview of the Fo-Fc omit map (contoured at 3.0 σ) around the inhibitor pocket for the CVB3 3Dpol–GPC-N143 complex. The inhibitor contacting residues in the polymerase binding pocket are indicated. (TIF) [file ppat.1004733.s008.tif]

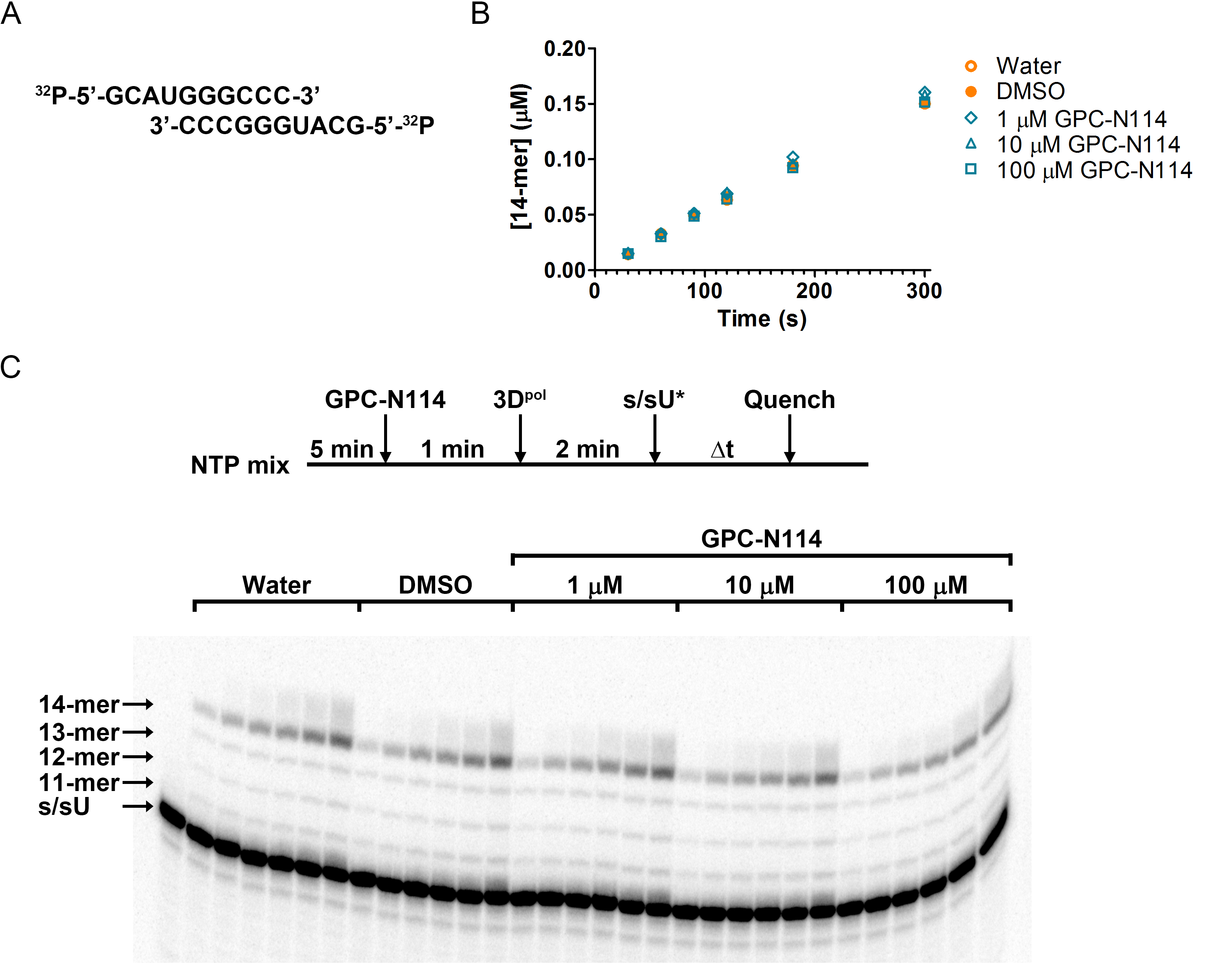

Supplement: S9 Fig — (A) The sequence of the sym/sub template primer duplex. (B-C) GPC-N114 has no effect on NTP incorporation in the sym/sub-U assay. NTPs (500 μM) were incubated in a buffer containing 50 mM Tris pH 7.0, 10 mM KCl, and 0.8 mM MgCl2 for 5 min. GPC-N114 or DMSO were added and the mix was incubated for another minute, followed by addition of 1 μM CVB3 3Dpol. After a two-minute incubation, the reaction was initiated with [32P]-labeled sym/sub-u (1 μM) and quenched at 30, 60, 90, 120, 180 and 300s after the initiation. Reaction products were analyzed by electrophoresis on a denaturing polyacrylamide gel (C). The quantification of the incorporation of NTPs is depicted in (B). (TIF) [file ppat.1004733.s009.tif]

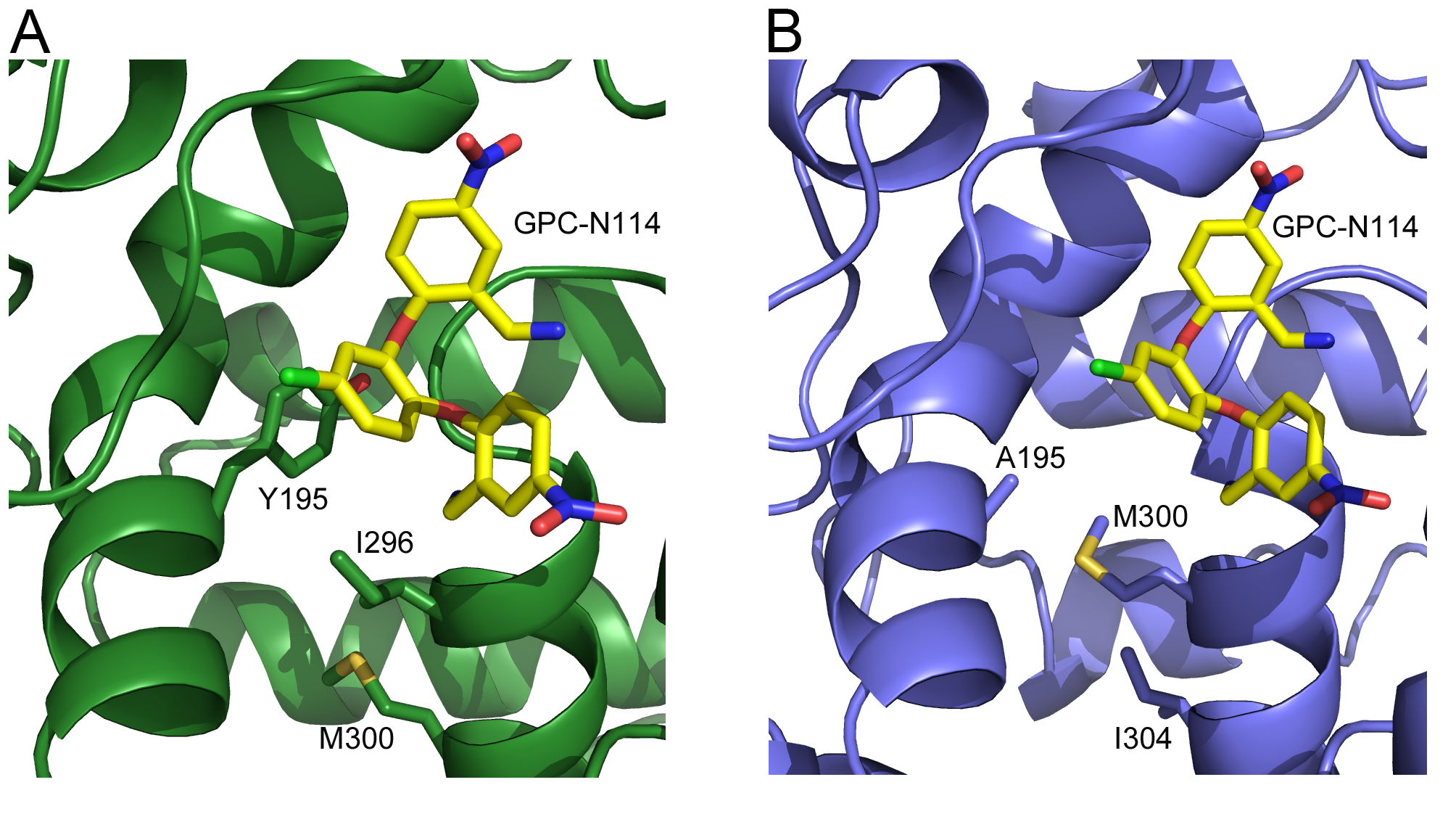

Supplement: S10 Fig — Cartoon representation of the GPC-N114-binding pocket in CVB3 3Dpol (forest green) (A) and the putative inhibitor pocket in EMCV (slate blue) (B). Polymerase side chains are represented in sticks only for: i) Y195 in CVB3 3Dpol, making crucial interactions with the compound, and its equivalent (A195) in EMCV 3Dpol, ii) the EMCV residues M300 and I304 that are mutated in GPC-N114-resistant mutants and the equivalent amino acids in CVB3 3Dpol (I296 and M300). The inhibitor is represented in atom-type sticks with carbon atoms in yellow. (TIF) [file ppat.1004733.s010.tif]
